# Feasibility and efficacy of chemoradiotherapy for elderly patients with locoregionally advanced nasopharyngeal carcinoma: results from a matched cohort analysis

**DOI:** 10.1186/1748-717X-8-70

**Published:** 2013-03-22

**Authors:** Huai Liu, Qiu-Yan Chen, Ling Guo, Lin-Quan Tang, Hao-Yuan Mo, Zong-Liang Zhong, Pei-Yu Huang, Dong-Hua Luo, Rui Sun, Xiang Guo, Ka-Jia Cao, Ming-Huang Hong, Hai-Qiang Mai

**Affiliations:** 1 State Key Laboratory of Oncology in South China, Sun Yat-sen University Cancer Center, Guangzhou, P.R. China; 2 Department of Nasopharyngeal Carcinoma, Sun Yat-sen University Cancer Center, 651 Dongfeng Road East, Guangzhou 510060, P.R. China; 3 Good Clinical Practice (GCP) Center, Sun Yat-sen University Cancer Center, Guangzhou, P.R. China

**Keywords:** Nasopharyngeal carcinoma, Elderly, Chemoradiotherapy, Adult comorbidity evaluation-27

## Abstract

**Background:**

To clarify the feasibility and efficacy of chemoradiotherapy (CRT) in elderly (age≥65 years) patients with locoregionally advanced nasopharyngeal carcinoma (NPC).

**Methods:**

From January 2000 to December 2006, 101 newly diagnosed elderly non-metastatic NPC patients (age≥65 years) who received cisplatin 3-weekly or weekly concurrent CRT with/without sequential chemotherapy were recruited. Each patient from the CRT group was matched to another patient treated with radiotherapy (RT) alone based on age, gender, pathological type, performance status, overall stage, stage method, Adult Comorbidity Evaluation-27 (ACE-27) score and RT technique, from the same institute and time period. We also recruited 101 young patients (age<65 years) as the referent group, which had been matched to the CRT group based on patient characteristics and treatment parameters. Treatment tolerability and toxicity were clarified, and treatment outcomes were calculated and compared among groups.

**Results:**

CRT was feasible in elderly NPC patients, while a concurrent regimen of weekly cisplatin was more tolerable. Grade≥3 acute toxicity in CRT group was similar with referent group, although it was significantly higher than the RT alone group (65.3% vs. 43.6%, *P*=0.002). Furthermore, patients with ACE-27 score≥2 in the CRT group had significantly higher severe acute toxicity and dose reduction. Survival was poorer in elderly patients than the referent group. Compared to RT alone, CRT significantly improved the 5-year overall survival (OS: 54.6% vs. 39.3%, *P*=0.009), cancer-specific survival (CSS: 56.6% vs. 42.7%, *P*=0.022), disease-free survival (DFS: 51.6% vs. 30.2%, *P*=0.028) and locoregional relapse-free survival (LRRFS: 78.4% vs. 52.2%, *P*=0.003), but not distant metastasis-free survival (DMFS: 69.6% vs. 63.6%, *P*=0.669). However, CRT did not significantly improve 5-year OS (43.6% vs. 27.3%, *P*=0.893) or CSS (43.6% vs. 34.1%, *P*=0.971) in elderly NPC patients with ACE-27 score≥2.

**Conclusions:**

CRT is feasible and effective in elderly patients with locoregionally advanced NPC without severe comorbidities. CRT should be used under serious consideration and be further tested in elderly patients with severe comorbidities. As such, it is essential to perform a comprehensive evaluation of pretreatment comorbidity status for all elderly NPC patients.

## Background

Nasopharyngeal carcinoma (NPC) differs from other head and neck cancers because of its unique characteristics with regard to epidemiology, pathological types and therapeutic managements [1]. NPC is prevalent in southern China, Singapore and Malaysia, although it is rare in the United States and Western Europe. The Intergroup Study 0099 and several other prospective randomized trials that were conducted in endemic areas have demonstrated that, compared to radiotherapy (RT) alone, concurrent chemoradiotherapy (CRT) with/without adjuvant chemotherapy could significantly improve survival of locoregionally advanced NPC, although with higher rates of acute toxicities [2-7]. Since then, concurrent CRT with/without adjuvant chemotherapy has become the standard treatment modality for these patients. However, elderly NPC patients only comprise a small part in these clinical trials because of restrictive selection criteria. In addition, in real clinical practice, co-existing ailments and decreasing organ function are common among the elderly. As such, the safety and efficacy of standard treatment modalities for the elderly population is still unknown. A previous systemic review showed that patients with comorbidities had a higher rate of chemotherapy-induced grade 3 to 4 toxicity [8]. When faced with the choice between benefits and toxicities caused by CRT, many oncologists prefer RT alone, which was already demonstrated to be tolerable in elderly patients with head and neck cancers and NPC [9-11], however, this conservative treatment selection may prevent some elderly patients from longer survival. Since the safety and outcome of CRT in elderly NPC patients are still not clear, the blind selection of either RT alone or CRT is inappropriate. Therefore, we conducted a matched cohort analysis to analyze the feasibility and efficacy of CRT in elderly locoregionally advanced NPC patients.

## Methods

### Patient population

This retrospective matched cohort analysis recruited elderly patients (aged≥65 years) with locoregionally advanced NPC treated by CRT or RT alone. The study was approved by the institutional reviewed board at Sun Yat-sen University Cancer Center. Selection criteria for CRT cases included the following patients: the elderly (age≥65 years); those with newly diagnosed NPC without metastasis; those with stage III-IVb disease (the 7th American Joint Committee on Cancer/Union for International Cancer Control (AJCC/UICC) staging system) ([12], Additional file 1), where patients assessed with a former staging system were re-staged according to the 7^th^ AJCC/UICC staging system; those who finished radical RT in the end; those treated with concurrent chemotherapy using cisplatin with/without sequential chemotherapy (e.g., neoadjuvant and/or adjuvant chemotherapy); and those without history of previous chemotherapy or RT.

**Figure 1:**
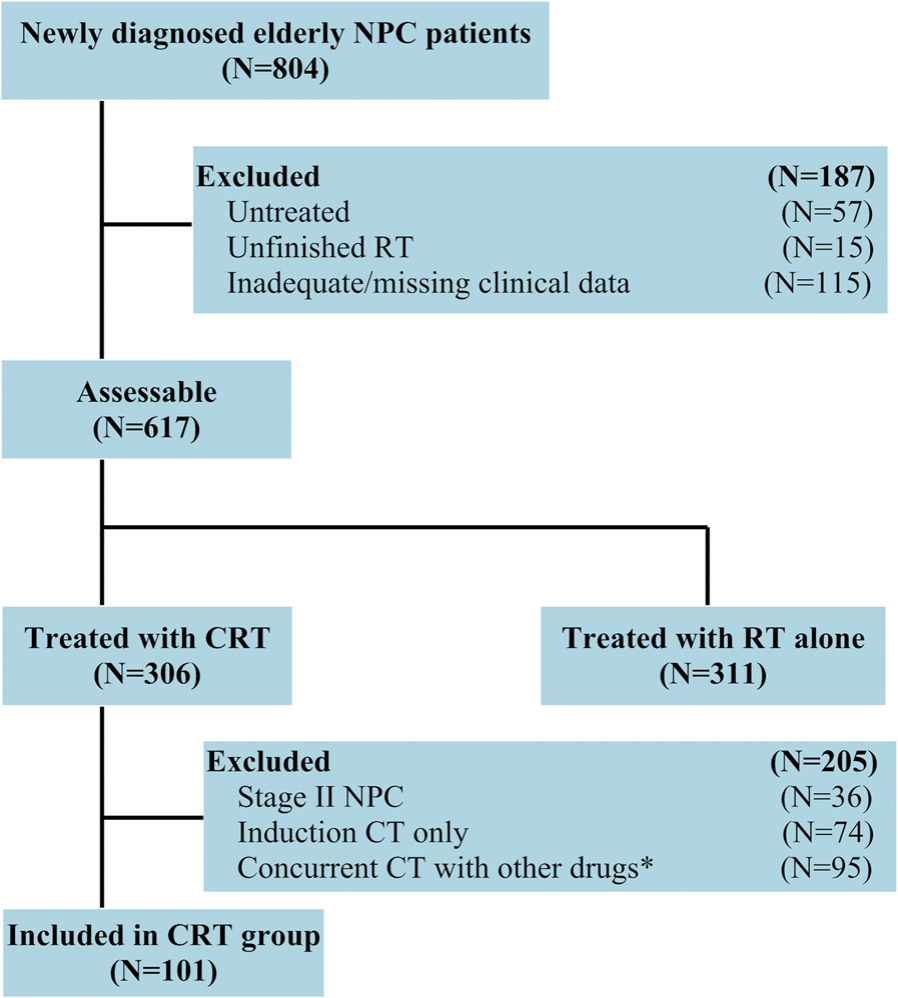
**Flowchart of patients.** NPC: Nasopharyngeal carcinoma; RT: Radiotherapy; CRT: Chemoradiotherapy; CT: Chemotherapy. *Other drugs included carboplatin (N=43), nedaplatin (N=28), paclitaxel (N=9), docetaxel (N=8), and Xeloda (N=7).

From January 2000 to December 2006, 11,173 newly diagnosed non-metastatic NPC patients were registered at Sun Yat-sen University Cancer Center, of them 804 (7.2%) patients were 65 years old or over. Figure 1 shows the flowchart of patients. Finally, 101 patients who met the above criteria were included in our CRT group. Each patient in the CRT group was matched with another patient from a group of 311 patients treated with RT alone at the same institution and during the same period. The matched prognostic factors included the following: age (65–69 years and ≥70 years), gender (male and female), pathological type (WHO types II-III and WHO type I), performance status (ECOG scores of 0–1 and 2), overall stage (stage III and IVa-b), stage method (CT and MRI), Adult Co-morbidity Evaluation-27 (ACE-27) ([13]; Additional file 2) scores (0–1 and 2–3) and RT techniques (2D conventional RT and 3D conformal RT or intensity-modulated radiotherapy (IMRT)). When an exact match was not available, a patient with more favorable characteristics from the RT alone group was selected to prevent results from being biased in favor of the CRT group. We also recruited 101 young patients (age<65 years) with locoregionally advanced NPC at the same institute and during the same time period as the referent group, and patient characteristics and treatment parameters were matched to the CRT group.

Table 1 summarizes the patient characteristics of the three groups. Elderly patients from the CRT and RT alone group were completely matched for age, gender, pathological type, ACE-27 score, and stage method. The percentages of patients matched for performance status, overall stage and RT technique were 99.0%, 97.0% and 99.0%, respectively. Except for ACE-27 score (98.0%) and RT technique (98.0%), patient characteristics were completely matched between the CRT and referent group.

**Table 1:** Patients’ characteristics

	**RT group (%)**	**CRT group (%)**	**Referent group (%)**
	**N=101**	**N=101**	**N=101**
**Age**			
≥65, <70	78 (77.2)	78 (77.2)	
≥70	23 (22.8)	23 (22.8)	
Median	68	69	45
Range	65-80	65-77	18-65
**Gender**			
Male	81 (80.2)	81 (80.2)	81 (80.2)
Female	20 (19.8)	20 (19.8)	20 (19.8)
**Histology**			
WHO type I	1 (1.0)	1 (1.0)	1 (1.0)
WHO type II	10 (9.9)	14 (13.9)	12 (11.9)
WHO type III	90 (89.1)	86 (85.1)	88 (87.1)
**PS score (ECOG)**			
0	0 (0)	1 (1.0)	1 (1.0)
1	101 (100)	99 (98.0)	99 (98.0)
2	0 (0)	1 (1.0)	1 (1.0)
**ACE-27 score**			
0	64 (63.4)	67 (66.3)	70 (69.3)
1	26 (25.7)	23 (22.8)	22 (21.8)
2	10 (9.9)	10 (9.9)	8 (7.9)
3	1 (1.0)	1 (1.0)	1 (1.0)
**Stage method**			
CT	37 (36.6)	37 (36.6)	37 (36.6)
MRI	64 (63.4)	64 (63.4)	64 (63.4)
T stage^*^			
T1-2	19 (18.8)	26 (25.7)	23 (22.8)
T3-4	82 (81.2)	75 (74.3)	78 (75.2)
N stage^*^			
N0-1	66 (65.3)	46 (45.5)	53 (52.5)
N2-3	35 (34.7)	55 (54.5)	48 (47.5)
Overall stage^*^			
III	50 (49.5)	47 (46.5)	47 (46.5)
IVa-b	51 (50.5)	54 (53.5)	54 (53.5)
**RT technique**			
2D conventional RT	93 (92.1)	94 (93.1)	92 (91.1)
3D conformal RT	1 (1.0)	1 (1.0)	1 (1.0)
IMRT	7 (6.9)	6 (5.9)	8 (7.9)
**RT dose (median)**			
NP	70	70	70
LN	60	62	60

Abbreviations: *WHO*, World Health Organization; *PS*, Performance Status; *ECOG*, Eastern Cooperative Oncology Group; *ACE-27*, Adult Comorbidity Evaluation-27; *CT*, Computed Tomography; *MRI*, Magnetic Resonance Imaging; *RT*, Radiotherapy; *IMRT*, Intensity-Modulated Radiotherapy; *NP*, Nasopharynx; *LN*, Lymph node.

^*^The 7th AJCC/UICC staging system.

### Pretreatment assessment

All patients were evaluated with a physical examination, complete blood count, liver and renal biochemistries, fiberoptic endoscopy of the nasopharynx, chest radiograph, abdominal ultrasonography, magnetic resonance imaging (MRI) or computed tomography (CT) of the nasopharynx and neck region, and bone scan by emission computed tomography.

We re-staged all patients using the 7^th^ AJCC/UICC staging system according to data from the clinical examinations and imaging.

ACE-27 was used as uniform criteria to evaluate comorbidity status. We reviewed individual medical records of each patient to perform the ACE-27 assessments. Comorbid conditions and ailments were categorized as none (score 0), mild (score 1), moderate (score 2), or severe (score 3), according to the degree of organ decompensation. Following principles described in the Comorbidity Coding Book [Additional file 3], those comorbidities lacking specific information were categorized as mild. These scores were then used to designate an overall comorbidity ranking according to the highest ranked ailment. Patients with more than two moderate ailments in different organ systems were considered as severe. Table 2 shows the details of comorbidity in two elderly groups. The most frequently affected systems were cardiovascular (17.8%), and then the gastrointestinal (11.9%) and endocrine systems (6.4%). Of note, 9 (4.5%) patients bore diseases from two or more organ systems.

**Table 2:** Comorbid grades and affected systems of elderly patients

Body system^*^	**N=202 (%)**	**RT alone (N=101)**			**CRT (N=101)**		
		**Mild**	**Moderate**	**Severe**	**Mild**	**Moderate**	**Severe**
Cardiovascular	36 (17.8)	11	5	1	13	6	0
Respiratory	3 (1.5)	2	0	0	1	0	0
Gastrointestinal	24 (11.9)	13	4	1	5	1	0
Endocrine	13 (6.4)	4	2	1	1	4	1
Neurological	3 (1.5)	0	1	0	1	1	0
Substance abuse	4 (2.0)	1	0	0	3	0	0
Multi-Systems	9 (4.5)	4	2	1	0	2	0
Overall	71 (35.1)	26	10	1	23	10	1

^*^Abnormality in other systems (renal, psychiatric, rheumatologic, immunological systems and malignancy, body weight) was not found in both groups.

### RT

All patients received radical radiotherapy with a 6 MV photon beam. In total, 92.6% (187/202) of elderly patients received conventional 2D RT, while only 1.0% (2/202) and 6.4% (13/202) received 3D conformal RT and IMRT, respectively. Patients were treated with conventional 2D RT using a uniform RT protocol based on the treatment policy for NPC at our cancer center [14].

RT was given five times a week at 2 Gy/day (2D conventional and 3D conformal RT) or 2.12-2.19 Gy/day (IMRT). The cumulative dose was 68–72 Gy to the primary tumor site, 60–62 Gy to the involved areas of the neck, and 50 Gy to uninvolved neck areas. The median cumulative dose of the primary site and neck area were 70 Gy and 60 Gy in RT alone group, 70 Gy and 62 Gy in CRT group, and 70 Gy and 60 Gy in the reference group, respectively.

### Chemotherapy

Patients from the CRT group received concurrent chemotherapy with cisplatin with/without sequential chemotherapy (neoadjuvant and or adjuvant). Cisplatin was administered at 80 mg/m^2^ 3-weekly or at 40 mg/m^2^ weekly. Selection of sequential chemotherapy and concurrent regimens were under consideration of individual doctor. Dose modification was performed, if necessary, at the discretion of the doctors.

Chemotherapy was not given in some patients because of rejected by patients and family, age over 75 years, participation in clinical trial, and presence of other medical conditions that were contra-indicative.

### Patient evaluation and follow-up

Patients were assessed at the time of treatment completion, at least once every three months over the next three years and at least once every six months thereafter. The patient evaluation included a clinical examination, nasopharyngeal endoscopy, CT or MRI of the nasopharynx and the neck area, chest radiograph and abdominal ultrasonography.

### Statistical methods

The following endpoints were examined: 5-year overall survival (OS), cancer-specific survival (CSS), disease-free survival (DFS), locoregional relapse-free survival (LRRFS), and distant metastasis-free survival (DMFS). All endpoints were calculated from the date of treatment commencement to the date of the observed endpoints or to the date of the last follow-up.

Category variables were compared using Pearson χ^2^ test. Survival endpoints were analyzed using the Kaplan-Meier product-limit method [15]. The log-rank test was used to analyze the statistical significance of differences among the survival curves [16]. Treatment toxicity rates between the two groups were compared using the chi-squared test (or Fisher’s exact test, if indicated).

Analyses were performed using the SPSS statistical software package, version 16.0 (SPSS, Chicago, IL, USA). All statistical tests were two-tailed, and *P*-values < .05 were considered to be statistically significant.

## Results

### Treatment compliance

Only 7 elderly patients (3.5%) experienced RT interruption, among whom 4 and 3 patients were from the CRT and RT groups, respectively. However, the difference between the groups did not show statistical significance (4.0% vs. 3.0%, Fisher’s exact test, *P*=1.000).

Table 3 lists the specifics of chemotherapy in the CRT and referent group. All patients received concurrent cisplatin chemotherapy, with 45 (44.6%) and 56 (55.4%) in the CRT group, and 51 (50.5%) and 50 (49.5%) patients in the referent group, receiving the 3-weekly and weekly regimen respectively (*P*=0.398). The chemotherapy cycles were similar in both groups (*P*=1.000 for 3-weekly regimen and *P*=0.956 for weekly regimen). In the CRT group, only 31.1% (14/45) of patients received three cycles of 3-weekly regimen. With respect to the weekly regimen, 58.9% of patients received at least six cycles of chemotherapy. There were 21 (20.8%) and 4 (4.0%) patients in the CRT and referent group received decreased doses of concurrent cisplatin (*P*<0.001). However, the median total dose of concurrent cisplatin was the same in both groups (240 mg/m^2^ for weekly regimen and 160 mg/m^2^ for 3-weekly regimen). Altogether, there were 27 (26.7%) patients in the CRT and referent group received sequential chemotherapy.

**Table 3:** Chemotherapy details

**Characteristics**	**CRT group**			**Referent group**		
	**NAC (%) N=15**	**CC (%) N1=45, N2=56**	**AC (%) N=12**	**NAC (%) N=15**	**CC (%) N1=51, N2=50**	**AC (%) N=12**
**Cycles**						
3-weekly regimen						
1	8 (53.3)	5 (11.1)	7 (58.3)	6 (40.0)	5 (9.8)	7 (58.3)
2	7 (46.7)	26 (57.8)	5 (41.7)	9 (60.0)	30 (58.8)	5 (41.7)
3	0 (0.0)	14 (31.1)	0 (0.0)	0 (0.0)	16 (31.4)	0 (0.0)
Weekly regimen						
5	0 (0)	23 (41.1)	0 (0)	0 (0)	20 (40.0)	0 (0)
6	0 (0)	22 (39.3)	0 (0)	0 (0)	19 (38.0)	0 (0)
7	0 (0)	11 (19.6)	0 (0)	0 (0)	11 (22.0)	0 (0)
**Dose modification**	3 (20.0)	21 (20.8)	4 (33.3)	2 (13.3)	4 (4.0)	2 (16.7)

Abbreviations: *NAC*, Neo-adjuvant chemotherapy; *CC*, Concurrent chemoerapy; *AC*, Adjuvant chemotherapy.

We further analyzed the relationship between chemotherapy tolerance and ACE-27 score in the CRT group. Totally, 21 patients had decreased chemotherapy dose, and we found 6 of them had ACE-27 score≥2, which was significant higher than patients with ACE-27 score<2 (54.5% vs. 16.7%, *P*=0.009).

### Toxicity

Incidences of major acute and late toxicities are listed in Table 4. The toxicities were graded using the Common Terminology Criteria for Adverse Events (CTCAE) v3.0 [17] or Radiation Therapy Oncology Group/European Organisation for Research and Treatment of Cancer (RTOG/EORTC) Late Radiation Morbidity Scoring Schema [18]. Altogether, 110 (54.5%) patients experienced one or more severe acute toxicities (grade≥3), although no treatment-related death was reported in either elderly group. However, patients in the CRT group had a significantly higher rate of any severe toxicities (65.3% in the CRT group vs. 43.6% in the RT group, *P*=0.002). Regarding non-hematologic toxicities, incidences of severe oral mucositis (52.5% vs. 37.6%, *P*=0.034) and severe emesis (6.0% vs. 0%, *P*=0.029) were significantly higher in the CRT group, while no significant difference was detected with regard to the incidence of severe dermatitis between the groups (12.9% in the CRT group vs. 7.9% in the RT group, *P*=0.249). No severe diarrhea and renal toxicity were seen in either group. Regarding hematologic toxicities, incidences of severe leukopenia (11.9% vs. 0%, *P*<0.001), and granulocytopenia (16.8% vs. 0%, *P*<0.001) were significantly higher in the CRT group. Late toxicities were also assessed in our study, which involved the following four conditions: xerostomia, ear problems, subcutaneous fibrosis and trismus. In total, the incidence rate of any severe late toxicity was 11.9% (24/202). Unlike with acute toxicity, the incidence of severe toxicity was comparable between both groups (10.1% in the CRT group vs. 12.9% in the RT group, *P*=0.664). In the referent group, 63 (62.4%) and 18 (17.8%) patients experienced grade≥3 acute and late toxicity, respectively. It is noteworthy that no significant difference was found in severe toxicity between the CRT and referent group (*P*=0.660 for acute toxicity and *P*=0.160 for late toxicity).

**Table 4:** Patients experienced severe (grade 3 or 4) toxicities by treatment group

**Toxicity**	**CRT (%, N=101)**			**RT (%, N=101)**			**P**
	**Grade 3**	**Grade 4**	**All**	**Grade 3**	**Grade 4**	**All**	
**Acute toxicity**							
Leukopenia	12 (11.9)	0 (0)	12 (11.9)	0 (0)	0 (0)	0 (0)	< 0.001
Granulocytopenia	17 (16.8)	0 (0)	17 (16.8)	0 (0)	0 (0)	0 (0)	< 0.001
Thrombocytopenia	5 (5.0)	0 (0)	5 (5.0)	0 (0)	0 (0)	0 (0)	0.059
Anemia	6 (5.9)	0 (0)	6 (5.9)	1 (1.0)	0 (0)	1 (1.0)	0.118
Mucositis	52 (51.5)	1 (1.0)	53 (52.5)	38 (37.6)	0 (0)	38 (37.6)	0.034
Dermatitis	13 (12.9)	0 (0)	13 (12.9)	8 (7.9)	0 (0)	8 (7.9)	0.249
Hearing loss	1 (1.0)	0 (0)	1 (1.0)	0 (0)	0 (0)	0 (0)	1.000
Emesis	6 (6.0)	0 (0)	6 (6.0)	0 (0)	0 (0)	0 (0)	0.029
Hepatitis	2 (2.0)	0 (0)	2 (2.0)	2 (2.0)	0 (0)	2 (2.0)	1.000
Total any	65 (64.4)	1 (1.0)	66 (65.3)	44 (43.6)	0 (0)	44 (43.6)	0.002
**Late toxicity**							
Xerostomia	2 (2.0)	0 (0)	2 (2.0)	2 (2.0)	0 (0)	2 (2.0)	1.000
Ear (deafness/otitis)	8 (8.0)	0 (0)	8 (8.0)	9 (8.9)	0 (0)	9 (8.9)	0.800
Subcutaneous Fibrosis	6 (6.0)	0 (0)	6 (6.0)	8 (7.9)	0 (0)	8 (7.9)	0.580
Trismus	3 (3.0)	0 (0)	3 (3.0)	1 (1.0)	0 (0)	1 (1.0)	0.621
Total any	11 (10.1)	0 (0)	11 (10.1)	13 (12.9)	0 (0)	13 (12.9)	0.664

Table 5 shows the severe toxicity in different ACE-27 scores in elderly patients. We found that the rate of severe acute toxicities was higher in patients with ACE-27 score≥2, but the difference did not reach statistical significance (68.2% vs. 52.8%, *P*=0.171). However, stratified analysis shows that patients with ACE-27 score≥2 in the CRT group had significantly higher rate of severe acute toxicities than patients with ACE-27 score<2 (100% vs. 61.1%, *P*=0.008), while the difference was not significant in the RT group (36.4% vs. 44.4%, *P*=0.752). For late toxicities, the severe toxicities were similar in different ACE-27 scores (data not shown).

**Table 5:** Association of severe acute toxicity and ACE-27 score

**Acute toxicity**	**Overall**			**RT group**			**CRT group**		
	**ACE-27≥2**	**ACE-27<2**	***P***	**ACE-27≥2**	**ACE-27<2**	***P***	**ACE-27≥2**	**ACE-27<2**	***P***
**Grade≥3**	15	95	0.171	4	40	0.752	11	55	0.008
**Grade<3**	7	85		7	50		0	35	

### Clinical response

The tumor response was evaluated by endoscopy and imaging at 3 months after RT. The complete response (CR) rate at the primary site was 96.0% (97/101) for elderly patients treated with CRT and 95.0% (96/101) for patients treated with RT alone. Similarly, the CR rate at the neck area was 93.1% (94/101) for elderly patients that received CRT and 92.1% (93/101) for patients received RT alone. In each elderly group, 90 patients (89.1%) had a complete response at both the primary site and neck area (*P*=1.000). For the referent group, the overall CR rate was 96.0% (97/101), and the CR rate at the primary site and neck area was 97.0% (98/101) and 98.0% (99/101), respectively. There was no significant difference in overall CR rate between the CRT and referent group (*P*=0.060).

### Survival

**Figure 2:**
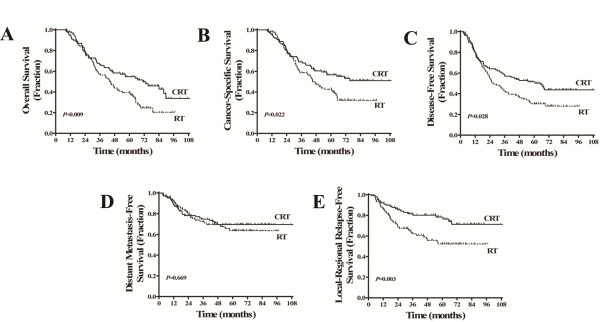
**Kaplan-Meier estimates of the survival of elderly locoregionally advanced nasopharyngeal carcinoma patients, by treatment group (CRT vs. RT).** (**A**) overall survival; (**B**) cancer-specific survival; (**C**) disease-free survival; (**D**) distant metastasis-free survival; and (**E**) locoregional relapse-free survival.

Our median follow-up period was 67.5 months (range: 8 to 114 months). One hundred and twenty-one deaths (52 from the CRT group vs. 69 from the RT group) were detected during the follow-up period, of which 104 (86.0%) were disease-related (44 in the CRT group vs. 57 in the RT group). Other causes of death included comorbidity (12 patients), old age (3 patients) and accidents (2 patients). The 5-year OS in the CRT group was 54.6% compared with 39.3% in the RT group (*P*=0.009, Figure 2A), with a hazard ratio of 0.619 (95% CI, 0.430-0.890; *P*=0.009). Thus, CRT decreased nearly 40% death risk in elderly locoregionally advanced NPC patients. Patients in the CRT group also had significantly higher 5-year CSS than did patients in the RT group (56.6% vs. 42.7%, *P*=0.022, Figure 2B). In the referent group, the 5-year OS and CSS were significantly higher than the CRT group (75.3% vs. 54.6%, *P*<0.001 and 77.8% vs. 56.6% *P*<0.001).

### Patterns of treatment failure

In total, 120 (59.4%) patients displayed progressed disease after treatment, among which 53 and 67 patients were from the CRT and RT groups, respectively. The 5-year DFS of the CRT group was higher than that of the RT group (51.6% vs. 30.2%, *P*=0.028, Figure 2C). Distant metastasis was common in our study. Altogether, 28.2% (57/202) of patients developed distant metastasis (28 and 29 patients in the CRT and RT groups, respectively). The 5-year DMFS was similar for both groups (69.6% in the CRT group vs. 63.6% in the RT group, *P*=0.669, Figure 2D). A total of 63 patients had local and/or regional relapse, among whom 22 and 41 patients were from the CRT and RT groups, respectively. Of note, patients from the CRT group had a significantly higher 5-year LRRFS (78.4% vs. 52.2%, *P*=0.003, Figure 2E). The 5-year DFS, and LRRFS were significantly higher in the referent group than the CRT group (68.2% vs. 51.6%, *P*=0.001 and 88.4% vs. 78.4%, *P*=0.017), but the difference in 5-year DMFS was not significant between both groups (78.2% vs. 69.6%, *P*=0.128).

### Efficacy in patients with severe comorbid status

We separated all elderly patients into two groups according to their comorbidity status (ACE-27 scores of 0–1 vs. 2–3). Based on a previous study that showed that elderly NPC patients with ACE-27 scores of 2–3 had poorer 5-year OS and CSS [10], we selected patients with severe comorbidity (ACE-27 scores of 2–3) to further explore the efficacy of CRT in this cohort.

**Figure 3:**
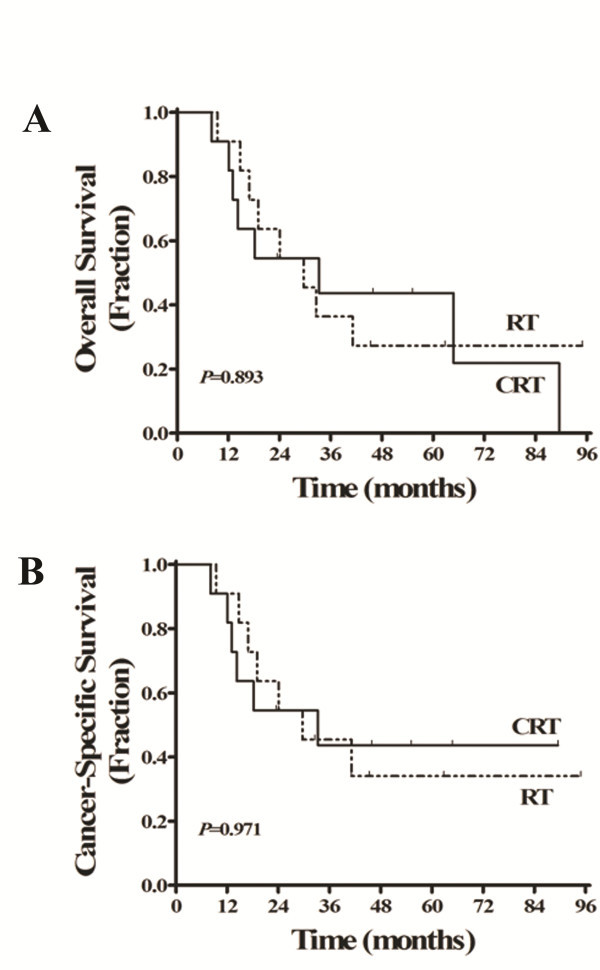
**Kaplan-Meier estimates of the survival of elderly locoregionally advanced nasopharyngeal carcinoma patients with severe comorbidities (ACE-27 score of 2–3), by treatment group (CRT vs. RT).** (**A**) overall survival and (**B**) cancer-specific survival.

Altogether, there were only 11 patients in each group. It is noteworthy that CRT did not improve either OS or CSS in these patients (5-year OS of 43.6% in the CRT group vs. 27.3% in the RT group, *P*=0.893, Figure 3A; 5-year CSS of 43.6% in the CRT group vs. 34.1% in the RT group, *P*=0.971; Figure 3B).

## Discussion

Three previous meta-analyses have confirmed that the addition of chemotherapy to radiotherapy could significantly improve survival in locoregionally advanced NPC patient, with concurrent chemotherapy displaying the largest effect [19-21]. Therefore, the National Comprehensive Cancer Network (NCCN) guidelines recommend concurrent CRT with adjuvant chemotherapy as the standard regimen for locoregionally advanced NPC patients. However, because elderly NPC patients who meet the strict selection criteria accounted for only a small part of these studies, the real role of chemotherapy in this population is still unclear. Our matched cohort analysis aimed to clarify the feasibility and efficacy of CRT among elderly locoregionally advanced NPC patients.

Combined chemotherapy and radiotherapy seemed to be less acceptable in the elderly NPC patients. Our data showed that many patients could not tolerate the full cycles of chemotherapy, with several patients experiencing dose modifications due to severe gastrointestinal reactions, suppressed bone marrow function and subjective denial. Regarding concurrent chemotherapy, only 31.1% of patients finished three cycles of high dose cisplatin, which was much lower than other published clinical trials (52%-71%) [2,7,22]. However, a weekly cisplatin regimen seemed to be much more tolerable, with 58.9% of patients completing at least 6 cycles in our study. Although, this result was lower than our previous phase III trial for stage II NPC patients (78.4%) [23], it is higher than those published by Chan et al. [24], which showed that only 44% of patients could tolerate at least 6 cycles of weekly cisplatin. According to clinical trials published to date, there have not been clear differences between the 3-weekly and weekly concurrent regimens of cisplatin in NPC. In present study, we found that the median total cisplatin dose in concurrent phase of weekly regimen was higher than that of 3-weekly regimen (240 mg/m^2^ vs. 160 mg/m^2^) in elderly patients. Therefore, the weekly concurrent regimen might be more appropriate for them.

The rates of severe acute and late toxicities in our study were similar to those of previously published clinical trials [2,5-7,23], and also we found that rates of severe toxicities caused by CRT in elderly patients were similar with young patients. A recent article that investigated multi-agent concurrent CRT for locally advanced head and neck squamous cell cancers in the elderly also found that toxicities were similar between the elderly and younger populations, although the elderly experienced more myelosuppression, required more unplanned hospitalization, and needed longer time with the feeding tube [25]. Furthermore, in our study, toxicities in both groups were manageable, with no incidence of treatment-related death and only a few cases of radiotherapy interruption. Therefore, combined CRT may be feasible in the elderly NPC population. We also found patients with severe comorbid status (ACE-27 score of 2–3) were related with significantly higher rate of severe acute toxicity in CRT group, but not in RT alone group. This is confirmed by previous review which showed comorbidity was related with higher grade 3 to 4 toxicity induced by chemotherapy [8].

Compared to other large clinical trials (which involved only a few elderly patients), the elderly patients in our study seemed to have poorer survival. The 5-year OS of the CRT and RT groups was 67% vs. 37% in the Intergroup Study 0099 [26]; 68% vs. 64% in the Hong Kong 9901 trial [5]; and 70.3% vs. 56.8% in another trial from Hong Kong [27], respectively. However, our results were 54.6% vs. 39.3%, respectively. With respect to 5-year CSS, the results in our study were also lower than theirs. Results were similar when the CRT group compared with the referent group. Previous studies have also reported poor treatment outcomes in elderly NPC patients [10,28,29]. Decreased body function, higher rates of co-existing comorbidities and inadequate tolerability for chemotherapy were potential reasons for the relatively poor treatment outcomes.

A meta-analysis that included 93 randomized trials and 17,246 head and neck patients (without any NPC patients) found that, although the addition of chemotherapy for locoregional treatment could significantly improve survival, the efficacy of chemotherapy decreased with increasing age (test for trend, *P*=0.003). The same report ascribed this phenomenon to the increased proportion of death not due to head and neck cancers with age (15% in patients younger than 50 years vs. 39% in patients 71 years and older) [30]. However, a subsequent retrospective study showed controversial results. Compared to patients<70 years, patients≥70 years with locally advanced head and neck squamous cell cancers treated with multi-agent concurrent CRT had nearly identical projected 5-year disease-specific survival (74% vs. 71%) and freedom from recurrence (71% vs. 69%) [25]. Regarding NPC, a meta-analysis of eight randomized trials and 1,753 locally advanced NPC patients detected no significant interactions between the treatment effect and age [20]. However, this analysis separated all patients into only three age groups (<41, 41–50 and ≥51), without exact numbers of elderly patients. Ho et al. used modified weekly cisplatin (30 mg/m^2^) as concurrent regimen in elderly NPC patients and still showed favorable 2-year survival, with OS, DFS, LRRFS and DMFS observed at 87%, 73%, 92% and 76%, respectively [31], but their sample was relatively small (26 patients could be analyzed) and it took patients aged lower than 65 years into analysis. In our study, we found that CRT could still significantly improve 5-year OS, CSS, DFS and LRRFS but not DMFS when compared to RT alone in elderly NPC patients. It is obvious that the benefits in OS and CSS were mainly derived from a higher locoregional control in the CRT group. Therefore, CRT is still important for elderly locoregionally advanced NPC patients.

However, CRT was not effective in all elderly patients. Our stratified study showed that CRT could not significantly improve either the 5-year OS or CSS in elderly NPC patients with severe comorbidities (ACE-27 scores of 2–3). Over 50% patients (ACE-27 score≥2) had dose reduction in the CRT group could be one of the important reason. A systemic review also showed that comorbidity was related to decreased chemotherapy use, tolerability and inferior survival in various cancers [8]. Considering the worse tolerance, higher severe acute toxicity and no significant survival benefit, using CRT in elderly NPC patients with severe comorbidity should be under serious consideration.

There are some limitations to this study. First of all, this is a retrospective study; secondly, our stratified study had a small sample; then, no quality of life data in this study; at last most of our patients were treated with 2D conventional RT, while the widely using of IMRT showed excellent locoregional control in NPC. Therefore, further prospective randomized trials with large sample should be conducted to test CRT in elderly NPC patients treated with IMRT.

## Conclusions

In conclusion, to the best of our knowledge, our study is the first one which directly compared CRT and RT alone in elderly NPC patients to explore feasibility and efficacy of CRT in them. We found that CRT was feasible and effective in elderly patients with locoregionally advanced NPC without severe comorbidities. CRT should be used under serious consideration and be further tested in elderly patients with severe comorbidities. As such, it is essential to perform a comprehensive evaluation of pretreatment comorbidity status for all elderly patients with NPC.

## Abbreviations

CRT: Chemoradiotherapy; NPC: Nasopharyngeal carcinoma; RT: Radiotherapy; ACE-27: Adult comorbidity evaluation-27; OS: Overall survival; CSS: Cancer-specific survival; DFS: Disease-free survival; LRRFS: Locoregional relapse-free survival; DMFS: Distant metastasis-free survival; AJCC: American joint committee on cancer; UICC: Union for international cancer control; IMRT: Intensity-modulated radiotherapy; MRI: Magnetic resonance imaging; CT: Computed tomography; CTCAE: Common terminology criteria for adverse events; RTOG: Radiation therapy oncology group; EORTC: European organisation for research and treatment of cancer; NCCN: National comprehensive cancer network.

## Competing interests

The authors have declared no conflicts of interest.

## Authors’ contributions

Guarantors of integrity of the entire study: HL, Q-YC, H-QM; study concepts/study design: H-QM; data acquisition: XG, K-JC, M-HH; data analysis/interpretation: all authors; literature review: H-YM, Z-LZ, P-YH, D-HL, RS; statistical analyses: HL, Q-YC, LG; manuscript drafting or revision for important intellectual content: HL, Q-YC, H-QM; manuscript final version approval: All authors read and approved the final manuscript.

## Supplementary Material

Additional file 1 The 7th edition of the ajcc/uicc staging system of nasopharyngeal carcinoma. Click here for file

Additional file 2 Adult comorbidity evaluation-27. Click here for file

Additional file 3 Comorbidity coding book. Click here for file
