# Supplementary material for: Feasibility and efficacy of chemoradiotherapy for elderly patients with locoregionally advanced nasopharyngeal carcinoma: results from a matched cohort analysis
Source: Radiat Oncol. 2013 Mar 22;8:70. doi: 10.1186/1748-717X-8-70 (PMC3643871; doi:10.1186/1748-717X-8-70)
Supplement: Additional file 1 — The 7th edition of the ajcc/uicc staging system of nasopharyngeal carcinoma. [file 1748-717X-8-70-S1.pdf]

The 7th edition of the AJCC/UICC staging system of nasopharyngeal carcinoma

**T1**	Tumor confined to the nasopharynx, or extends to oropharynx and/or nasal cavity without parapharyngeal extension
**T2**	Tumor with parapharyngeal extension
**T3**	Tumor involves bony structures of skull base and/or paranasal sinuses
**T4**	Tumor with intracranial extension and/or involvement of cranial nerves, hypopharynx, orbit, or with extension to the infratemporal fossa/masticator space
**N0**	No regional lymph node metastasis
**N1**	Unilateral metastasis in lymph node(s), 6 cm or less in greatest dimension, above the supraclavicular fossa, and/or unilateral or bilateral, retropharyngeal lymph nodes, 6 cm or less, in great dimension
**N2**	Bilateral metastasis in lymph node(s), 6 cm or less in greatest dimension, above the supraclavicular fossa
**N3**	Metastasis in a lymph node(s) >6 cm and/or extension to supraclavicular fossa
**N3a**	Greater than 6 cm in dimension
**N3b**	Extension to the supraclavicular fossa
**M0**	No distant metastases
**M1**	Distant metastases Present
**Stage I**	T1N0M0

**Stage II**	T2N0-1M0 to T1-2N1M0
**Stage III**	T3N0-2M0 to T1-3N2M0
**Stage IVA**	T4N0-2M0
**Stage IVB**	Any T N3 M0
**Stage IVC**	Any T Any N M1
