# Supplementary material for: Feasibility and efficacy of chemoradiotherapy for elderly patients with locoregionally advanced nasopharyngeal carcinoma: results from a matched cohort analysis
Source: Radiat Oncol. 2013 Mar 22;8:70. doi: 10.1186/1748-717X-8-70 (PMC3643871; doi:10.1186/1748-717X-8-70)
Supplement: Additional file 2 — Adult comorbidity evaluation-27. [file 1748-717X-8-70-S2.pdf]

Adult Comorbidity Evaluation-27

Identify the important medical comorbidities and grade severity using the index.
Overall Comorbidity Score is defined according to the highest ranked single ailment, except in the case where two or more Grade 2 ailments occur in different organ systems. In this situation, the overall comorbidity score should be designated Grade 3.

Cogent comorbid ailment	Grade 3<br>Severe Decompensation	Grade 2<br>Moderate Decompensation	Grade 1<br>Mild Decompensation
**Cardiovascular System**			
Myocardial Infarct	☐ MI ≤ 6 months	☐ MI > 6 months ago	☐ MI by ECG only, age undetermined
Angina / Coronary Artery Disease	☐ Unstable angina	☐ Chronic exertional angina<br>☐ Recent (≤ 6 months) Coronary Artery Bypass Graft (CABG) or Percutaneous Transluminal Coronary Angioplasty (PTCA)<br>☐ Recent (≤ 6 months) coronary stent	☐ ECG or stress test evidence or catheterization evidence of coronary disease without symptoms<br>☐ Angina pectoris not requiring hospitalization<br>☐ CABG or PTCA (>6 mos.)<br>☐ Coronary stent (>6 mos.)
Congestive Heart Failure (CHF)	☐ Hospitalized for CHF within past 6 months<br>☐ Ejection fraction < 20%	☐ Hospitalized for CHF >6 months prior<br>☐ CHF with dyspnea which limits activities	☐ CHF with dyspnea which has responded to treatment<br>☐ Exertional dyspnea<br>☐ Paroxysmal Nocturnal Dyspnea (PND)
Arrhythmias	☐ Ventricular arrhythmia ≤ 6 months	☐ Ventricular arrhythmia > 6 months<br>☐ Chronic atrial fibrillation or flutter<br>☐ Pacemaker	☐ Sick Sinus Syndrome<br>☐ Supraventricular tachycardia
Hypertension	☐ DBP≥130 mm Hg<br>☐ Severe malignant papilledema or other eye changes<br>☐ Encephalopathy	☐ DBP 115-129 mm Hg<br>☐ DBP 90-114 mm Hg while taking antihypertensive medications<br>☐ Secondary cardiovascular symptoms: vertigo, epistaxis, headaches	☐ DBP 90-114 mm Hg while <u>not</u> taking antihypertensive medications<br>☐ DBP <90 mm Hg while taking antihypertensive medications<br>☐ Hypertension, not otherwise specified
Venous Disease	☐ Recent PE (≤ 6 mos.)<br>☐ Use of venous filter for PE's	☐ DVT controlled with Coumadin or heparin<br>☐ Old PE > 6 months	☐ Old DVT no longer treated with Coumadin or Heparin
Peripheral Arterial Disease	☐ Bypass or amputation for gangrene or arterial insufficiency < 6 months ago<br>☐ Untreated thoracic or abdominal aneurysm (≥6 cm)	☐ Bypass or amputation for gangrene or arterial insufficiency > 6 months ago<br>☐ Chronic insufficiency	☐ Intermittent claudication<br>☐ Untreated thoracic or abdominal aneurysm (< 6 cm)<br>☐ s/p abdominal or thoracic aortic aneurysm repair
**Respiratory System**			
	☐ Marked pulmonary insufficiency<br>☐ Restrictive Lung Disease or COPD with dyspnea at rest despite treatment<br>☐ Chronic supplemental O_2<br>☐ CO_2 retention (pCO_2 > 50 torr)<br>☐ Baseline pO_2 < 50 torr<br>☐ FEV1 (< 50%)	☐ Restrictive Lung Disease or COPD (chronic bronchitis, emphysema, or asthma) with dyspnea which limits activities<br>☐ FEV1 (51%-65%)	☐ Restrictive Lung Disease or COPD (chronic bronchitis, emphysema, or asthma) with dyspnea which has responded to treatment<br>☐ FEV1 (66%-80%)
**Gastrointestinal System**			
Hepatic	☐ Portal hypertension and/or esophageal bleeding ≤ 6 mos. (Encephalopathy, Ascites, Jaundice with Total Bilirubin > 2)	☐ Chronic hepatitis, cirrhosis, portal hypertension with moderate symptoms "compensated hepatic failure"	☐ Chronic hepatitis or cirrhosis without portal hypertension<br>☐ Acute hepatitis without cirrhosis<br>☐ Chronic liver disease manifested on biopsy or persistently elevated bilirubin (>3 mg/dl)
Stomach / Intestine	☐ Recent ulcers(≤ 6 months ago) requiring blood transfusion	☐ Ulcers requiring surgery or transfusion > 6 months ago	☐ Diagnosis of ulcers treated with meds<br>☐ Chronic malabsorption syndrome<br>☐ Inflammatory bowel disease (IBD) on meds or h/o with complications and/or surgery
Pancreas	☐ Acute or chronic pancreatitis with major complications (phlegmon, abscess, or pseudocyst)	☐ Uncomplicated acute pancreatitis<br>☐ Chronic pancreatitis with minor complications (malabsorption, impaired glucose tolerance, or GI bleeding)	☐ Chronic pancreatitis w/o complications

Cogent comorbid ailment	Grade 3<br>Severe Decompensation	Grade 2<br>Moderate Decompensation	Grade 1<br>Mild Decompensation
**Renal System**			
End-stage renal disease	☐ Creatinine > 3 mg% with multi-organ failure, shock, or sepsis<br>☐ Acute dialysis	☐ Chronic Renal Insufficiency with creatinine >3 mg%<br>☐ Chronic dialysis	☐ Chronic Renal Insufficiency with creatinine 2-3 mg%.
**Endocrine System**	**(Code the comorbid ailments with the (*) in both the Endocrine system and other organ systems if applicable)**		
Diabetes Mellitus	☐ Hospitalization ≤ 6 months for DKA<br>☐ Diabetes causing end-organ failure<br>☐ retinopathy<br>☐ neuropathy<br>☐ nephropathy*<br>☐ coronary disease*<br>☐ peripheral arterial disease*	☐ IDDM without complications<br>☐ Poorly controlled AODM with oral agents	☐ AODM controlled by oral agents only
**Neurological System**			
Stroke	☐ Acute stroke with significant neurologic deficit	☐ Old stroke with neurologic residual	☐ Stroke with no residual<br>☐ Past or recent TIA
Dementia	☐ Severe dementia requiring full support for activities of daily living	☐ Moderate dementia (not completely self-sufficient, needs supervising)	☐ Mild dementia (can take care of self)
Paralysis	☐ Paraplegia or hemiplegia requiring full support for activities of daily living	☐ Paraplegia or hemiplegia requiring wheelchair, able to do some self care	☐ Paraplegia or hemiplegia, ambulatory and providing most of self care
Neuromuscular	☐ MS, Parkinson’s, Myasthenia Gravis, or other chronic neuromuscular disorder and requiring full support for activities of daily living	☐ MS, Parkinson’s, Myasthenia Gravis, or other chronic neuromuscular disorder, but able to do some self care	☐ MS, Parkinson’s, Myasthenia Gravis, or other chronic neuromuscular disorder, but ambulatory and providing most of self care
**Psychiatric**			
	☐ Recent suicidal attempt<br>☐ Active schizophrenia	☐ Depression or bipolar disorder uncontrolled<br>☐ Schizophrenia controlled w/ meds	☐ Depression or bipolar disorder controlled w/ medication
**Rheumatologic**	**(Incl. Rheumatoid Arthritis, Systemic Lupus, Mixed Connective Tissue Disorder, Polymyositis, Rheumatic Polymyositis)**		
	☐ Connective Tissue Disorder with secondary end-organ failure (renal, cardiac, CNS)	☐ Connective Tissue Disorder on steroids or immunosuppressant medications	☐ Connective Tissue Disorder on NSAIDS or no treatment
**Immunological System**	**(AIDS should not be considered a comorbidity for Kaposi's Sarcoma or Non-Hodgkin's Lymphoma)**		
AIDS	☐ Fulminant AIDS w/KS, MAI, PCP (AIDS defining illness)	☐ HIV+ with h/o defining illness. CD4^{+} < 200/μL	☐ Asymptomatic HIV+ patient.<br>☐ HIV^{+} w/o h/o AIDS defining illness. CD4^{+} > 200/μL
**Malignancy**	**(Excluding Cutaneous Basal Cell Ca., Cutaneous SCCA, Carcinoma in-situ, and Intraepithelial Neoplasm)**		
Solid Tumor including melanoma	☐ Uncontrolled cancer<br>☐ Newly diagnosed but not yet treated<br>☐ Metastatic solid tumor	☐ Any controlled solid tumor without documented metastases, but initially diagnosed and treated within the last 5 years	☐ Any controlled solid tumor without documented metastases, but initially diagnosed and treated > 5 years ago
Leukemia and Myeloma	☐ Relapse<br>☐ Disease out of control	☐ 1st remission or new dx <1yr<br>☐ Chronic suppressive therapy	☐ H/o leukemia or myeloma with last Rx > 1 yr prior
Lymphoma	☐ Relapse	☐ 1st remission or new dx <1yr<br>☐ Chronic suppressive therapy	☐ H/o lymphoma w/ last Rx >1 yr prior
**Substance Abuse**	**(Must be accompanied by social, behavioral, or medical complications)**		
Alcohol	☐ Delirium tremens	☐ Active alcohol abuse with social, behavioral, or medical complications	☐ H/o alcohol abuse but not presently drinking
Illicit Drugs	☐ Acute Withdrawal Syndrome	☐ Active substance abuse with social, behavioral, or medical complications	☐ H/o substance abuse but not presently using
**Body Weight**			
Obesity		☐ Morbid (i.e., BMI ≥ 38)	

OVERALL COMORBIDITY SCORE (Circle one.)

0	1	2	3	9
**None**	**Mild**	**Moderate**	**Severe**	**Unknown**
