# Supplementary material for: Feasibility and efficacy of chemoradiotherapy for elderly patients with locoregionally advanced nasopharyngeal carcinoma: results from a matched cohort analysis
Source: Radiat Oncol. 2013 Mar 22;8:70. doi: 10.1186/1748-717X-8-70 (PMC3643871; doi:10.1186/1748-717X-8-70)
Supplement: Additional file 3 — Comorbidity coding book. [file 1748-717X-8-70-S3.pdf]

COMORBIDITY CODING BOOK

Prepared by:

Jay F. Piccirillo, MD, FACS
Amy Johnston, BS
Peter Claybour, BA
Benjamin Littenberg, MD
Cynthia Creech, CTR
Scott Anderson, CTR
Lori Grove, CTR
Irene Costas, BS, MPH

Washington University School of Medicine
Department of Otolaryngology – Head and Neck Surgery
Clinical Outcomes Research Office
Campus Box 8115
517 South Euclid Avenue
St. Louis, MO 63110
Academic Office: 314-362-7394
http://oto.wustl.edu/clinepi/outcomes.htm

Research supported in part by grants from the
National Cancer Institute
Cancer Education Program
https://www-commons.cit.nih.gov/crisp/

INTRODUCTION 3

SELECTED REFERENCES 4

GENERAL GUIDELINES FOR CODING COMORBIDITY 5

AMBIGUOUS TERMINOLOGY 8

ADULT COMORBIDITY EVALUATION-27 9

CODING OF INDIVIDUAL DISEASES, CONDITIONS, AND ILLNESSES 13

CARDIOVASCULAR SYSTEM 13
RESPIRATORY SYSTEM 18
GASTROINTESTINAL SYSTEM 20
RENAL SYSTEM 23
ENDOCRINE SYSTEM 24
NEUROLOGICAL SYSTEM 25
PSYCHIATRIC 27
RHEUMATOLOGIC 28
IMMUNOLOGICAL SYSTEM 29
MALIGNANCY 30
SUBSTANCE ABUSE 36
BODY WEIGHT 37

SCENARIOS 38

INDEX OF ABBREVIATIONS OF PROCEDURES AND DISEASES 54

PARTIAL DRUG LIST 59

INTRODUCTION

Patients with cancer often have other diseases, illnesses, or conditions in addition to their index cancer. These other conditions are generally referred to as comorbidities. Although not a feature of the cancer itself, comorbidity is an important attribute of the patient. Survival rates are lower for patients with a greater number and severity of comorbid conditions. Comorbidity also has direct impact on the care of patients, selection of initial treatment, and evaluation of treatment effectiveness. When reporting statistical survival data, hospital-based and national cancer registries do not routinely take into account these coexisting medical ailments.

The goal of this coding book is to assist in the education and training of Certified Tumor Registrars and other individuals dedicated to collecting and reporting information on patients with cancer. This book should be used in association with the videotape, "The Whole Picture: Coding Comorbidity," which may be obtained from the Clinical Outcomes Research Office. The coding book should also serve as a resource to answer questions and to guide continued accurate and valid collection of comorbid information. We are most interested in all comments from users of this coding book so we may improve this work for future users.

We are currently developing a web-based version of this education program. We believe this web-based version should be of great value in training cancer registrars and other individuals to code comorbidity from the medical records of patients with cancer. When completed (approximately April-May, 2003), this web course may be accessed through the Clinical Outcomes Research Office website http://oto.wustl.edu/clinepi.

Jay F. Piccirillo, MD
Benjamin Littenberg, MD
Cynthia Creech, CTR
Lori Grove, CTR
Amy Johnston, BS
Peter Claybour, BA
Irene Costas, BS, MPH

St. Louis, Missouri
September 2002

SELECTED REFERENCES

1. Piccirillo JF. Inclusion of Comorbidity in a Staging System for Head and Neck Cancer. *Oncology*. 1995 9(9):831-836.

2. Piccirillo JF, Feinstein AR. Clinical Symptoms and Comorbidity: Significance for the Prognostic Classification of Cancer. *Cancer*. 1996; 77:834-842.

3. Pugliano FA, Piccirillo JF. The Importance of Comorbidity in Staging Upper Aerodigestive Tract Cancer. *Current Opinion Otolaryngology Head Neck Surgery*. 1996; 4:88-93.

4. Piccirillo JF, Creech CM, Zequeira R, Anderson S, Johnston AS. Inclusion of Comorbidity into Oncology Data Registries. *Journal of Registry Management* 1999;26:66-70.

5. Piccirillo JF. Importance of Comorbidity in Head and Neck Cancer. *Laryngoscope*. 2000; 110(4):593-602.

6. Johnston, AS, Piccirillo JF, Creech C, Littenberg B, Jeffe D, Spitznagel EL. Validation of a Comorbidity Education Program. *Journal of Registry Management* 2001; 28: 125-131.

7. Piccirillo JF, Costas I, Claybour P, Borah AJ, Grove L. The Measurement of Comorbidity by Cancer Registries. *Journal of Registry Management* (accepted for publication)

GENERAL GUIDELINES FOR CODING COMORBIDITY

Definition

Comorbidity refers to any co-existing medical ailment not related to the index cancer. For example, in a patient with prostate cancer, congestive heart failure would be considered a comorbidity. Any medical ailment related to the cancer should not be considered comorbidity. For example, in a patient with hepatic carcinoma, portal hypertension with ascites would not be considered a comorbidity. Instead, the portal hypertension is a manifestation of the hepatic carcinoma. In this particular case, the patient has ascites because of portal hypertension related to the hepatic tumor and the ascites cannot be considered a comorbidity.

Time of Assessment

- Comorbidity will be classified based on the illness(es**) present at or before the date of diagnosis** (also referred to as **zero-time** in this document). Any other illness(es) referred to in the chart which are diagnosed after the diagnosis of the index cancer should not be considered comorbid condition(s). If a comorbid condition is detected from laboratory tests or diagnostic medical work-up during the hospital admission for diagnosis and/or initial treatment of the index cancer, code the comorbid condition as a comorbidity. If a complication of the cancer or its treatment occurs (e.g., post-operative myocardial infarction), do not code the complication as a comorbid condition.

Guidelines for Severity Score

- The degree of decompensation of **Individual** comorbid ailments should be classified as Grade 1 (Mild Decompensation), Grade 2 (Moderate Decompensation), or Grade 3 (Severe Decompensation).

- The **Overall Comorbidity Severity Score** (Mild, Moderate, or Severe) is defined according to the highest ranked single ailment, ***except*** when two or more Grade 2 ailments occur in different organ systems. In this case, the comorbid overall severity score should be designated as a **Severe**.

  For example, three Grade 1 illnesses in different organ systems (chronic bronchitis treated with medication, well-controlled hypertension, and diabetes controlled by oral agents alone) would still be coded as an Overall Comorbidity Score of Mild or Grade 1. On the other hand, congestive heart failure more than 6 months ago and portal hypertension without complications, (two Grade 2 comorbidities in different organ systems) would have an Overall Comorbidity Severity Score of Severe or Grade 3.

Key Points

- If a medical condition is not listed on the ACE-27 comorbidity form or in this coding book, do not code it as comorbidity.

  Examples: anemia and gout

- In the case of ambiguity between grades of comorbidity for a single condition, always select the least severe grade.

- If you know a comorbidity exists, however it does not exactly match the definition on the comorbidity form, it is important that you capture this information rather than exclude it because it does not fit perfectly.

  Example: Grade 1 Respiratory is defined as "Restrictive Lung Disease or COPD (chronic bronchitis, emphysema, or asthma) with dyspnea which has responded to treatment." The medical record states the patient has chronic emphysema but does not indicate whether there is dyspnea or whether there has been a response to treatment. In this case, you would indicate that the patient has a Grade one Respiratory comorbidity.

- Similarly, within a grade for a single condition, there may be two options given but you are unsure which option to check. In this case, rather than record neither comorbidity option, you would record the comorbidity which most closely matches the condition described in the medical record.

- Do not try to assign a comorbidity based solely on the medications that the patient is taking. Only code the comorbidity if the disease itself is mentioned in the medical record.

  Example: A patient may be taking an anti-depressant but without mention of depression. In this case, do not code **Depression**. The patient may be taking the medication for other reasons (e.g., to induce weight loss).

- Unless otherwise specified, the terms "acute" and "recent" refer to events less than or equal to six months prior to the time of diagnosis. The terms "chronic" and "old" refer to events greater than six months prior to the time of diagnosis.

Key Points, continued

- A transplant (kidney, liver, lung or heart) is <u>not</u> comorbidity. Rather, it is an indication of the severity AND irreversibility of the index medical condition.

  Examples: A 62 year old male hospitalized for congestive heart failure and subsequent heart transplant greater than 6 months prior to diagnosis would receive a Grade of 2 for "Hospitalized for CHF greater than 6 months prior." Similarly, a 59 year old female with acute kidney transplant rejection who is on dialysis at the time of diagnosis would receive a Grade of 2 for "Acute dialysis."

- Toxic Exposure. Exposure to toxic agents should not be coded as a comorbidity. Many toxic agents such as radiation, ethylene oxide, and asbestos have been implicated in the **pathogenesis** of human cancers. Unfortunately, it is usually difficult to quantify the exact amount of exposure and assess the relative contribution of such exposure to the existence of comorbid conditions in cancer patients.

AMBIGUOUS TERMINOLOGY

Terms That Constitute a Diagnosis of Comorbid Ailment

Interpret the following terms as a diagnosis of comorbid ailment.

- Compatible with
- Consistent with
- Most likely
- Presumably
- Probable
- Suspect
- Suspicious

Example: Medical record states: "CXR was consistent with COPD. Patient refused further work-up." "Patient's history and physical are compatible with a diagnosis of myocardial infarction pending test results."

Terms That Do Not Constitute a Diagnosis of Comorbid Ailment

Do not interpret the following terms as a diagnosis of comorbid ailment. Do not include patients who have a diagnosis consisting only of these terms.

- Equivocal
- Possible
- Questionable
- "Claims to be"
- Suggests
- Worrisome
- Rule Out

Example: Admitting diagnosis is reported as: "possible myocardial infarct."

ADULT COMORBIDITY EVALUATION-27

The Kaplan-Feinstein Index[1] (KFI) was originally developed by Morrison Kaplan and Alvan Feinstein in 1974. Drs. Jay Piccirillo and Benjamin Littenberg modified this instrument to form the Adult Comorbidity Evaluation-27. The KFI was modified for two important reasons. First, the KFI does not list diabetes as a comorbid ailment since the KFI was developed from the study of a cohort of patients with diabetes (index disease). Diabetes is a common condition and could impact on outcomes for patients with cancer and so it was included as a cogent comorbid ailment in the ACE-27. Second, the KFI did not include several other important conditions, such as deep vein thrombosis; heart, lung, liver, and kidney transplants; paraplegia; Multiple Sclerosis, Parkinson's disease, Myasthenia Gravis, dementia, schizophrenia, bipolar disorder, depression, AIDS, substance abuse, and morbid obesity. Drs. Piccirillo, Littenberg, and colleagues sought advice from clinical experts and, when possible, the published literature to assign levels of comorbidity to the ailments not included in the original instrument.

Validation of the ACE-27

The medical records of 190 patients of Barnes-Jewish Hospital were selected for detailed review and assessment of the performance of the ACE-27 comorbidity index. The patients were selected to ensure a broad representation of tumor sites and severity of comorbidity levels. Comorbidity, using the ACE-27, was classified without knowledge of the KFI score. The study population of 190 patients consisted of 98 (52%) women, 149 (78%) Caucasians, and the mean (SD) age was 64.3 (13.0). The overall two-year survival rate was 113/190 (60%).

The reviewers included (A) a first year medical student and (B) a trained research assistant from the Clinical Outcomes Research Office who trained registrars to code comorbidity for approximately one year. Of the 190 selected charts, Observer A reviewed all 190 while Observer B reviewed 112 charts twice, with a 5-month interval between reviews. Due to misclassification of the primary tumor site, three charts were excluded from the sample. The Weighted Kappa Statistic was used to assess interobserver and intraobserver reliability, and the statistical significance criterion was set at 0.05.

Significant agreement between the independent observers was noted with a Weighted Kappa of 0.80 (95%CI, 0.72,0.88). Intraobserver reliability was also significant with a Weighted Kappa of 0.93 (95%CI, 0.88,0.98). Kappa measurements which are greater than 0.75 indicate excellent reproducibility.

---

[1] Kaplan, Feinstein. *J Chron Dis.* 1974;27:387-404

Prognostic Validity of the ACE-27

Between the years of 1999-2001, the Clinical Outcomes Research Office has received 7,697 completed ACE-27 forms for newly diagnosed (analytic) cancer patients presenting at six nationwide participating medical centers. Of these forms, detailed demographic, tumor, and follow-up information has been electronically received from the participating medical centers for the first 3,326 patients.

The prognostic impact of comorbidity on survival is shown in Figure 1. As can be seen, there is a strong prognostic impact of comorbidity on survival (Log Rank χ^2 = 195.24; $p<0.0001$). At any point in time, patients with more severe levels of comorbidity have worse survival.

FIGURE 1: Impact of Comorbidity on Survival
N=3,326

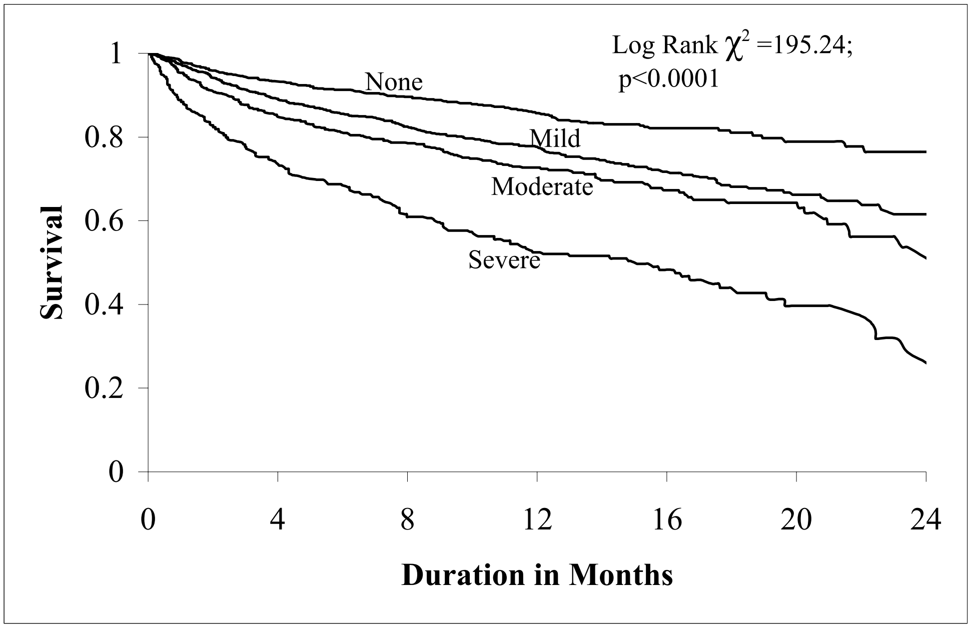

Date ____________________
Coder's Initials ____________
Oncology Center __________
Accession # _______________

Adult Comorbidity Evaluation-27

Identify the important medical comorbidities and grade severity using the index.
Overall Comorbidity Score is defined according to the highest ranked single ailment, except in the case where two or more Grade 2 ailments occur in different organ systems In this situation, the overall comorbidity score should be designated Grade 3.

Cogent comorbid ailment	Grade 3<br>Severe Decompensation	Grade 2<br>Moderate Decompensation	Grade 1<br>Mild Decompensation
**Cardiovascular System**			
Myocardial Infarct	☐ MI ≤ 6 months	☐ MI > 6 months ago	☐ MI by ECG only, age undetermined
Angina / Coronary Artery Disease	☐ Unstable angina	☐ Chronic exertional angina<br>☐ Recent (≤ 6 months) Coronary Artery Bypass Graft (CABG) or Percutaneous Transluminal Coronary Angioplasty (PTCA)<br>☐ Recent (≤ 6 months) coronary stent	☐ ECG or stress test evidence or catheterization evidence of coronary disease without symptoms<br>☐ Angina pectoris not requiring hospitalization<br>☐ CABG or PTCA (>6 mos.)<br>☐ Coronary stent (>6 mos.)
Congestive Heart Failure (CHF)	☐ Hospitalized for CHF within past 6 months<br>☐ Ejection fraction < 20%	☐ Hospitalized for CHF >6 months prior<br>☐ CHF with dyspnea which limits activities	☐ CHF with dyspnea which has responded to treatment<br>☐ Exertional dyspnea<br>☐ Paroxysmal Nocturnal Dyspnea (PND)
Arrhythmias	☐ Ventricular arrhythmia ≤ 6 months	☐ Ventricular arrhythmia > 6 months<br>☐ Chronic atrial fibrillation or flutter<br>☐ Pacemaker	☐ Sick Sinus Syndrome
Hypertension	☐ DBP≥130 mm Hg<br>☐ Severe malignant papilledema or other eye changes<br>☐ Encephalopathy	☐ DBP 115-129 mm Hg<br>☐ DBP 90-114 mm Hg while taking antihypertensive medications<br>☐ Secondary cardiovascular symptoms: vertigo, epistaxis, headaches	☐ DBP 90-114 mm Hg while <u>not</u> taking antihypertensive medications<br>☐ DBP <90 mm Hg while taking antihypertensive medications<br>☐ Hypertension, not otherwise specified
Venous Disease	☐ Recent PE (≤ 6 mos.)<br>☐ Use of venous filter for PE's	☐ DVT controlled with Coumadin or heparin<br>☐ Old PE > 6 months	☐ Old DVT no longer treated with Coumadin or Heparin
Peripheral Arterial Disease	☐ Bypass or amputation for gangrene or arterial insufficiency < 6 months ago<br>☐ Untreated thoracic or abdominal aneurysm (≥6 cm)	☐ Bypass or amputation for gangrene or arterial insufficiency > 6 months ago<br>☐ Chronic insufficiency	☐ Intermittent claudication<br>☐ Untreated thoracic or abdominal aneurysm (< 6 cm)<br>☐ s/p abdominal or thoracic aortic aneurysm repair
**Respiratory System**			
	☐ Marked pulmonary insufficiency<br>☐ Restrictive Lung Disease or COPD with dyspnea at rest despite treatment<br>☐ Chronic supplemental O_2<br>☐ CO_2 retention (pCO_2 > 50 torr)<br>☐ Baseline pO_2 < 50 torr<br>☐ FEV1 (< 50%)	☐ Restrictive Lung Disease or COPD (chronic bronchitis, emphysema, or asthma) with dyspnea which limits activities<br>☐ FEV1 (51%-65%)	☐ Restrictive Lung Disease or COPD (chronic bronchitis, emphysema, or asthma) with dyspnea which has responded to treatment<br>☐ FEV1 (66%-80%)
**Gastrointestinal System**			
Hepatic	☐ Portal hypertension and/or esophageal bleeding ≤ 6 mos. (Encephalopathy, Ascites, Jaundice with Total Bilirubin > 2)	☐ Chronic hepatitis, cirrhosis, portal hypertension with moderate symptoms "compensated hepatic failure"	☐ Chronic hepatitis or cirrhosis without portal hypertension<br>☐ Acute hepatitis without cirrhosis<br>☐ Chronic liver disease manifested on biopsy or persistently elevated bilirubin (>3 mg/dl)
Stomach / Intestine	☐ Recent ulcers (≤ 6 months ago) requiring blood transfusion	☐ Ulcers requiring surgery or transfusion > 6 months ago	☐ Diagnosis of ulcers treated with meds<br>☐ Chronic malabsorption syndrome<br>☐ Inflammatory bowel disease (IBD) on meds or h/o with complications and/or surgery
Pancreas	☐ Acute or chronic pancreatitis with major complications (phlegmon, abscess, or pseudocyst)	☐ Uncomplicated acute pancreatitis<br>☐ Chronic pancreatitis with minor complications (malabsorption, impaired glucose tolerance, or GI bleeding)	☐ Chronic pancreatitis w/o complications

Cogent comorbid ailment	Grade 3 Severe Decompensation	Grade 2 Moderate Decompensation	Grade 1 Mild Decompensation
**Renal System**			
End-stage renal disease	☐ Creatinine > 3 mg% with multi-organ failure, shock, or sepsis<br>☐ Acute dialysis	☐ Chronic Renal Insufficiency with creatinine >3 mg%<br>☐ Chronic dialysis	☐ Chronic Renal Insufficiency with creatinine 2-3 mg%.
**Endocrine System**	**(Code the comorbid ailments with the (*) in both the Endocrine system and other organ systems if applicable)**		
Diabetes Mellitus	☐ Hospitalization ≤ 6 months for DKA<br>☐ Diabetes causing end-organ failure<br>☐ retinopathy<br>☐ neuropathy<br>☐ nephropathy*<br>☐ coronary disease*<br>☐ peripheral arterial disease*	☐ IDDM without complications<br>☐ Poorly controlled AODM with oral agents	☐ AODM controlled by oral agents only
**Neurological System**			
Stroke	☐ Acute stroke with significant neurologic deficit	☐ Old stroke with neurologic residual	☐ Stroke with no residual<br>☐ Past or recent TIA
Dementia	☐ Severe dementia requiring full support for activities of daily living	☐ Moderate dementia (not completely self-sufficient, needs supervising)	☐ Mild dementia (can take care of self)
Paralysis	☐ Paraplegia or hemiplegia requiring full support for activities of daily living	☐ Paraplegia or hemiplegia requiring wheelchair, able to do some self care	☐ Paraplegia or hemiplegia, ambulatory and providing most of self care
Neuromuscular	☐ MS, Parkinson's, Myasthenia Gravis, or other chronic neuromuscular disorder and requiring full support for activities of daily living	☐ MS, Parkinson's, Myasthenia Gravis, or other chronic neuromuscular disorder, but able to do some self care	☐ MS, Parkinson's, Myasthenia Gravis, or other chronic neuromuscular disorder, but ambulatory and providing most of self care
**Psychiatric**			
	☐ Recent suicidal attempt<br>☐ Active schizophrenia	☐ Depression or bipolar disorder uncontrolled<br>☐ Schizophrenia controlled w/ meds	☐ Depression or bipolar disorder controlled w/ medication
**Rheumatologic**	**(Incl. Rheumatoid Arthritis, Systemic Lupus, Mixed Connective Tissue Disorder, Polymyositis, Rheumatic Polymyositis)**		
	☐ Connective Tissue Disorder with secondary end-organ failure (renal, cardiac, CNS)	☐ Connective Tissue Disorder on steroids or immunosuppressant medications	☐ Connective Tissue Disorder on NSAIDS or no treatment
**Immunological System**	**(AIDS should not be considered a comorbidity for Kaposi's Sarcoma or Non-Hodgkin's Lymphoma)**		
AIDS	☐ Fulminant AIDS w/KS, MAI, PCP (AIDS defining illness)	☐ HIV+ with h/o defining illness. $CD4^+ < 200/\mu L$	☐ Asymptomatic HIV+ patient.<br>☐ HIV^+ w/o h/o AIDS defining illness. $CD4^+ > 200/\mu L$
**Malignancy**	**(Excluding Cutaneous Basal Cell Ca., Cutaneous SCCA, Carcinoma in-situ, and Intraepithelial Neoplasm)**		
Solid Tumor including melanoma	☐ Uncontrolled cancer<br>☐ Newly diagnosed but not yet treated<br>☐ Metastatic solid tumor	☐ Any controlled solid tumor without documented metastases, but initially diagnosed and treated within the last 5 years	☐ Any controlled solid tumor without documented metastases, but initially diagnosed and treated > 5 years ago
Leukemia and Myeloma	☐ Relapse<br>☐ Disease out of control	☐ 1st remission or new dx <1yr<br>☐ Chronic suppressive therapy	☐ H/o leukemia or myeloma with last Rx > 1 yr prior
Lymphoma	☐ Relapse	☐ 1st remission or new dx <1yr<br>☐ Chronic suppressive therapy	☐ H/o lymphoma w/ last Rx >1 yr prior
**Substance Abuse**	**(Must be accompanied by social, behavioral, or medical complications)**		
Alcohol	☐ Delirium tremens	☐ Active alcohol abuse with social, behavioral, or medical complications	☐ H/o alcohol abuse but not presently drinking
Illicit Drugs	☐ Acute Withdrawal Syndrome	☐ Active substance abuse with social, behavioral, or medical complications	☐ H/o substance abuse but not presently using
**Body Weight**			
Obesity		☐ Morbid (i.e., BMI ≥ 38)	

OVERALL COMORBIDITY SCORE (Circle one.) **0** None **1** Mild **2** Moderate **3** Severe **9** Unknown

Rev May 2003

CODING OF INDIVIDUAL DISEASES, CONDITIONS, AND ILLNESSES

Cardiovascular System

Myocardial Infarction

Grade 1, Slight Decompensation
- MI as demonstrated by ECG with the age undetermined. The ECG is the only documentation of the previous asymptomatic MI.

Grade 2, Moderate Decompensation
- MI more than 6 months prior to zero time

Grade 3, Full Decompensation
- MI within the past 6 months of zero time

Angina/Coronary Artery Disease

Grade 1
- ECG or stress test evidence or catheterization evidence of coronary disease without symptoms
- Angina pectoris not requiring hospitalization
- Angina attack compensated with treatment
- Coronary Artery Bypass Graft (CABG) or Percutaneous Transluminal Coronary Angioplasty (PTCA) for severe Coronary Artery Disease (CAD) greater than 6 months prior to zero time
- Coronary stent placement greater than 6 months prior to zero time

Grade 2
- Chronic exertional angina
- Recent Coronary Artery Bypass Graft (CABG) or Percutaneous Transluminal Coronary Angioplasty (PTCA) for severe Coronary Artery Disease (CAD) within past 6 months of zero time
- Coronary stent placement within the past 6 months of zero time

Grade 3
- Unstable angina - (clinical syndrome characterized by angina of new onset, angina at rest or with minimal exertion, or a crescendo pattern of angina with episodes of increasing frequency, severity or duration). May carry a risk of acute myocardial infarction as high as 20%.

Congestive Heart Failure

Grade 1
- CHF with dyspnea which has responded to treatment
- Exertional dyspnea
- Paroxysmal Nocturnal Dyspnea (PND)

Grade 2
- Hospitalized for congestive heart failure (CHF) more than 6 moths prior to zero time
- Congestive heart failure (CHF) with dyspnea which limits activities

Grade 3
- Hospitalized for CHF within the past 6 months of zero time
- Ejection fraction of less than 20%.

Note: The ejection fraction can be found on the echocardiogram.

Arrhythmia

Grade 1
- Sick Sinus Syndrome (SSS)

Grade 2
- Ventricular arrhythmia more than 6 months prior to zero time
- Chronic atrial fibrillation or flutter (Please note new onset also coded as Grade 2)
- Pacemaker at the date of diagnosis regardless of time used

Grade 3
- Ventricular arrhythmia within the past 6 months of zero time

Note:
- Arrhythmia not otherwise specified, bradyarrhythmia, and sinus tachyarrhythmia (> 100 beats per minute) are not coded as comorbid conditions.
- Arrhythmic comorbid conditions should be coded regardless if being treated with medication
- Arrhythmic conditions (other than ventricular arrhythmia) must be present at the date of diagnosis to be coded
- Notation of Wolff-Parkinson-White Syndrome, Wenckebach, or other types of named syndromes associated with atrial fibrillation should be coded Grade 2 Chronic Atrial Fibrillation or Flutter

Mini Scenario: A man had a pacemaker 10 years ago for a short period of time, but he did not have the pacemaker nor did he have any arrhythmias at the time of diagnosis. Therefore, the individual would not be given a comorbidity score for the arrhythmia.

Hypertension

Note: On a blood pressure reading, the top number is the systolic blood pressure and the bottom number is the diastolic blood pressure.

Grade 1

- Diastolic blood pressure (DBP) between 90-114 mm Hg with diagnosed hypertension, not taking antihypertensive medication
- DBP <90 mm Hg while taking antihypertensive medications (See list of medications below Grade 3)
- Hypertension, not otherwise specified
- No secondary cardiovascular symptoms such as headaches, epistaxis, vertigo, edema, etc

Grade 2

- DBP between 115 - 129 mm Hg with diagnosed hypertension, regardless of treatment
- DBP between 90-114 mm Hg while taking antihypertensive medication
- Secondary cardiovascular symptoms: vertigo, epistaxis, headaches, etc., regardless of treatment

Grade 3

- DBP greater or equal to 130 mm Hg, regardless of treatment
- Severe malignant papilledema or other eye changes, such as retinal damage, retinal hemorrhages, and/or retinal exudates, regardless of treatment
- Encephalopathy, regardless of treatment

Note: Do not code hypertension as comorbidity based on single elevated DBP unless the patient is specifically diagnosed with hypertension. An elevated DBP recorded on a nursing note or anesthesia-screening examination may be related to stress and anxiety and should not be used solely to determine grade. On the other hand, consistently elevated DBP is to be coded. It is anticipated that most hypertensive patients will receive a severity grade of 1.

Venous Disease

Grade 1
- Old deep vein thrombosis (DVT) no longer treated with medication (Coumadin or Heparin)

Grade 2
- DVT controlled with Coumadin or Heparin
- Old pulmonary embolism (greater than 6 months prior to zero time)

Grade 3
- Recent PE (less than six months prior to zero time)
- Use of venous filter to prevent pulmonary embolism (PE) dissemination
- Patient required surgery (filter) and is now on anticoagulation therapy to prevent further thrombus formation

Peripheral Arterial Disease

Grade 1
- Intermittent claudication
- Untreated thoracic or abdominal aneurysm (< 6 cm)
- s/p abdominal or thoracic aortic aneurysm repair

Grade 2
- Bypass or amputation for gangrene or arterial insufficiency (e.g., carotid endarterectomy, femoral/popliteal bypass, etc.) greater than six months prior to diagnosis
- Chronic arterial insufficiency

Grade 3
- Bypass or amputation for gangrene or arterial insufficiency (e.g., carotid endarterectomy, femoral/popliteal bypass, etc.) less than six months prior to diagnosis
- Untreated thoracic or abdominal aneurysm ($\geq$6 cm)
- Acute unexpected arterial condition usually secondary to blood clot or other limited event

Note: Amputation must consist of whole foot or higher.

List of selected Cardiovascular Drugs

Ace Inhibitors
- lisinopril (Prinivil, Zestril)
- benazepril (Lotensin)
- captopril (Capoten)
- enalapril (Vasotec)
- fosinopril (Monopril)
- quinapril (Accupril)
- ramipril (Altace)

Angiotensin II Receptor Anatagonists
- losartan (Cozaar)
- valsartan (Diovan)
- irbesartan (Avapro)
- candesartan (Atacand)

Beta Blockers
- metoprolol (Lopressor)
- bisoprolol (Zebeta)
- propranolol (Inderal)
- carvedilol (Coreg)
- penbutolol (Levatol)

Diuretics
- furosemide (Lasix)
- bumetanide (Bumex)
- torsemide (Demadex)
- chlorothiazide (Diuril)

Digitalis
- Digoxin (Lanoxin)

Vasodilators
- hydralazine (Apresoline)
- isosorbide dinitrate

Aldosterone-receptor inhibitor
- spironolactone (Aldactone)

Calcium-channel blockers
- diltiazem (Cardizem, Dilacor, Diltia, Tiamate, Tiazac)
- verapamil (Calan, Ioptin, Verelan, Covera)
- amoldipine (Norvasc)
- nifedipine (Adalat, Procardia)

Respiratory System

COPD - clinical syndrome of chronic dyspnea with expiratory airflow obstruction due to chronic bronchitis, emphysema, and asthma.

Grade 1
- Patient with diagnosed Restrictive Lung Disease or COPD that has responded to treatment activity.
- Dyspnea with significant activity (flight of stairs) or dyspnea of episodic nature
- Forced Expiratory Volume (FEV1) (66% - 80%)

Grade 2
- Patient with diagnosed Restrictive Lung Disease or COPD with dyspnea which limits activities.
- Dyspnea on mild exertion (walking from chair to TV)
- FEV1 (51% - 65%)

Grade 3
- Marked pulmonary insufficiency.
- Restrictive Lung Disease or COPD with dyspnea at rest despite treatment
- Chronic supplemental oxygen
- Carbon dioxide retention (as evidenced by arterial carbon dioxide value of greater than 50 torr) ($pCO_2 > 50$ torr)
- Hypoxemia (low blood oxygen) as evidenced by PO2 < 50 torr
- FEV1 (<50%)

Note:
- 1 torr = 1 mmHg
- ALL applicable conditions should be checked on the ACE-27. For example, a person with a FEV1 = 49% and COPD with dyspnea at rest should have two boxes checked on the comorbidity form under Grade 3.
- If multiple information is given with different severity grading, select the least severe grade. See example below.

Mini Scenario: A 55 year old man has been diagnosed with lung cancer. He has a 3-year history of COPD and a 52 pack-year history. He is being treated with two respiratory medications. The medical record states "dyspnea on exertion which limits activities" and "SOB with short walks." Lab values show a FEV1 = 47%. The statements in the chart indicate moderate comorbidity, while the FEV1 value suggests severe comorbidity. Since multiple information is given, select **Moderate**, the least severe grade between the two.

List of selected Respiratory Drugs

Anti-Leukotrienes
• zafirlukast (Accolate)
• zileuton (Zyflo)
Theophylline (Slo-Bid Gyrocaps, Unidur, Theo-Dur)
Anticholinergic agents
• ipratropium (Combivent)
BRONCHODILATORS
β- Adrenergic agonists
• albuterol (Proventil, Ventolin)
• bitolterol mesylate (Tornalate)
• pirbuterol (Maxair)
• terbutaline (Brethaire)
• salmeterol (Serevent)
ANTI-INFLAMMATORY DRUGS
Corticosteroids
• beclomethasone dipropionate (Beclovent, Vanceril)
• budesonide (Pulmicort Turbuhaler)
• flunisolide (Aerobid)
• fluticasone propionate (Flovent)
• Triamcinolone acetonide (Azmacort)
• Prednisone
Cromolyn sodium (Intal)
Nedocromil sodium (Tilade)

Gastrointestinal System

Hepatic

Grade 1
• Chronic hepatitis or cirrhosis without portal hypertension
• Acute hepatitis (except type A) without cirrhosis
• Chronic active or carrier cases of hepatitis (except type A)
• Chronic liver disease manifested on biopsy or persistently elevated bilirubin (>3 mg/dl)

Grade 2
• Chronic hepatitis, cirrhosis, or portal hypertension with moderate symptoms (including hepatomegaly, cutaneous spiders, caput medusa, palmar erythema) as a manifestation of chronic liver disease or "compensated hepatic failure"

Grade 3
• Patient with portal hypertension and/or esophageal bleeding ≤ 6 mos. (Encephalopathy, Ascites, Jaundice with Total Bilirubin >2)

Note: Code all types of Hepatitis (B, C, D, E, and NANB), <u>except</u> Hepatitis A. If medical record only states "hepatitis in past" or "exposure to hepatitis", then do not code as a comorbidity.

Mini Scenario: A male was diagnosed with colorectal cancer in May of 1998. In the History and Physical portion of the medical record, there is a statement "Hepatitis 1983." Since the type of hepatitis is not specified and no other information is given, do not code hepatitis as a comorbid condition.

Stomach/Intestine

Grade 1
• Diagnosis of ulcers including peptic ulcer disease (PUD), duodenal ulcer, or erosive gastritis treated with medications only
• Chronic malabsorption syndrome.
• Chronic Inflammatory Bowel Diseases -- Ulcerative colitis (UC), Crohn's disease, or Inflammatory Bowel Disease (IBD) on medication or with history of complications and/or surgery due to this condition.

Note: Erosive gastritis includes NSAID gastroenteropathy, stress-related mucosal damage and ulcers, alcohol gastropathy, phlegmonous gastritis, chronic erosive gastritis, postoperative alkaline gastritis, gastric ischemia, Menetrier's disease, eosinophilic gastritis, granulomatous gastritis, watermelon stomach

Grade 2

- Diagnosis of ulcers, including PUD, duodenal ulcer, or erosive gastritis with bleeding requiring surgery or transfusion greater than 6 months ago.

Grade 3

- Diagnosis of ulcers, including PUD, duodenal ulcer, or erosive gastritis with recent (≤ 6 months) major bleeding.

Note: Gastroesophageal reflux alone or history of ulcers, but not currently on medications, should not be coded as comorbid conditions.

List of Selected Drugs for Treatment of Inflammatory Bowel Diseases
Ulcerative colitis

- Sulfasalazine
- Corticosteroids
- Immunosuppressive agents (Azathioprine (AZT), 6-mercaptopurine)

Crohn's disease

- Sulfasalazine
- Corticosteroids
- Immunosuppressive agents (Azathioprine (AZT), 6-mercaptopurine)
- Metronidazole

List of Selected Drugs for Treatment of Ulcers
Antibacterials

- bismuth subslicylate (Pepto Bismol)
- tetracycine (Achromycin)
- metronidazole (Flagyl)
- amoxicillin (Amoxil)
- clarithromycin (Biaxin)

H_2-receptor antagonists

- cimetidine (Tagamet)
- famotidine (Pepcid)
- nizatidine (Axid)
- ranitidine (Zantac)

Omeprazole (Prilosec)
Sucralfate (Carafate)
Misoprostol (Cytotec)
Magnesium hydroxide / aluminum hydroxide / simethicone antacids (Maalox Plus, Mylanta Double Strength)

Pancreas

Grade 1

- Chronic pancreatitis (abnormal lab values especially Lipase and Amylase) without complications.

Grade 2

- Uncomplicated acute pancreatitis.
- Chronic pancreatitis with minor complications (e.g., malabsorption, impaired glucose tolerance, GI bleeding, icterus, bone pain, or endocrine insufficiency)

Grade 3

- Acute or chronic pancreatitis with major complications (e.g., infection, pseudocyst formation, pulmonary complications, acute renal failure, abscess, or phlegmon)

Renal System

Grade 1
• Chronic renal insufficiency with Creatinine 2-3 mg %.

Grade 2
• Chronic renal insufficiency with Creatinine > 3 mg % without secondary effects
• Chronic dialysis (on dialysis for more than 6 months prior to zero time)

Note: Secondary effects include anemia (low hematocrit), acidosis, hypertension, renal osteodystrophy (bone disease), secondary hyperparathyroidism (increase parathyroid hormone) due to hyperphosphatemia (elevated phosphate) and hypocalcemia (low calcium), and hypermagnesemia.

Grade 3
• Creatinine greater than 3 mg% with multi-organ failure, shock, or sepsis
• End stage renal disease (ESRD) receiving dialysis
• Acute dialysis (on dialysis less than 6 months prior to zero time)

Note: Do not code kidney transplant as comorbidity. Code the underlying medical condition.

Mini Scenario: A woman had a kidney transplant two years prior to her cancer diagnosis. The kidney was rejected immediately after the transplant occurred. She has been on dialysis ever since the rejection. She would receive a **2** for **chronic dialysis**.

Endocrine System

Diabetes Mellitus (DM)

Grade 1
• Adult onset diabetes mellitus (AODM) controlled by oral agents

Grade 2
• Insulin-dependent diabetes without complications or end-organ failure
• AODM poorly controlled with oral agents. Poorly controlled implies elevated glucose values despite use of oral agents. Frequent medical visits for evaluation of blood glucose and modifications in therapy.

Grade 3
• Diabetic ketoacidosis (DKA) with recent hospitalization.
• Diabetes causing end organ failure including retinopathy, neuropathy, nephropathy, coronary disease, and peripheral arterial disease.

Note:
• If AODM is controlled by diet alone do not code as a comorbid condition.
• For Grade 3, code the comorbid ailments of nephropathy, coronary disease, and peripheral arterial disease in both the endocrine system and other organ system (i.e., Renal, Cardiovascular, or Peripheral Vascular Disease)

Mini Scenario 1: A man is diagnosed with pancreatic cancer and has a "recent" diagnosis of diabetes. The chart states that the date of diagnosis for the diabetes is 2 months prior to the diagnosis of cancer. The patient is on oral meds at the time of cancer diagnosis. Since the diabetes occurred because of the disease process of the pancreatic cancer, do not code the diabetes as a comorbidity.

Mini Scenario 2: A woman has had IDDM for several years prior to a diagnosis of cancer. The chart states that she has retinopathy treated with laser eye surgery. The chart did not specifically state that the retinopathy is associated with the IDDM. Because diabetes mellitus is the leading cause of retinopathy, especially in those with known longstanding diabetes mellitus, code this case as diabetes causing retinopathy – Grade 3.

Neurological System

Stroke or Cerebral Vascular Accident (CVA)

Grade 1
- Old (greater than 6 months) stroke without neurological residual
- Past transient ischemic attacks (TIAs) or reversible ischemic neurological deficit (RIND)

Note: TIA- transient ischemic attacks that are focal and abrupt in onset usually lasting for 5-20 minutes and which may last as long as 24 hrs.
RIND- reversible ischemic neurologic deficit lasts longer than 24 hrs. and less than 7 days

Grade 2
- Old stroke with residual

Grade 3
- Acute stroke with coma or significant neurologic deficit

Note: Residual includes loss of vision, difficulty speaking, aphasia (loss or impairment of the power to use or comprehend words), paresis, or sensory disturbance

Dementia

Grade 1
- Mild dementia (can take care of self).

Grade 2
- Moderate dementia (not completely self-sufficient, needs supervision)

Grade 3
- Severe dementia (Confined to nursing home or needs constant support.)

Note: Basic Activities of Daily Living (ADL)
Eating, Bathing, Toileting, Dressing, Grooming,
Transferring (to/from bed/chair/bath), Ambulating (or other locomotion), and Communicating
Instrumental Activities of Daily Living (IADL)
Writing, Reading, Cooking, Cleaning, Shopping, Doing laundry, Climbing stairs, Using the telephone, Managing medications, Managing money, Traveling out of home, Maintaining upkeep of home

Paralysis

Grade 1
- Paraplegia or hemiplegia but ambulatory and providing most of self care

Grade 2
- Paraplegia or hemiplegia requiring wheelchair, but able to do some self care

Grade 3
- Paraplegia or hemiplegia requiring full support for activities of daily living

Neuromuscular

Grade 1
- Multiple Sclerosis, Parkinson's disease, Myasthenia Gravis, or other chronic neuromuscular disorder, but ambulatory and providing most of self care

Grade 2
- Multiple Sclerosis, Parkinson's disease, Myasthenia Gravis, or other chronic neuromuscular disorder, but able to do some self care

Grade 3
- Multiple Sclerosis, Parkinson's disease, Myasthenia Gravis, or other chronic neuromuscular disorder and requiring full support for activities of daily living

Psychiatric

Grade 1
• Depression or bipolar disorder controlled w/ medication

Grade 2
• Depression or bipolar disorder uncontrolled
• Schizophrenia controlled with medication

Grade 3
• Recent suicide attempt
• Active schizophrenia

Note: Depression is not situational and should predate the diagnosis of cancer.
Anxiety is not considered a comorbid condition.

Rheumatologic

Connective Tissue Diseases including Rheumatoid Arthritis, Systemic Lupus Erythematosus (SLE), Mixed connective tissue disease (MCTD), Polimyositis (PM), Rheumatic Polymyositis, Dermatomyositis (DM), Scleroderma

Note: Osteoarthritis (Degenerative Joint Disease), fibromyalgia, and carpal tunnel syndrome are not coded as comorbid conditions

Grade 1

• Connective Tissue Disorder on non-steroidal anti-inflammatory drugs (NSAIDS) or no treatment

Grade 2

• Connective Tissue Disorder on steroids or immunosuppressant medications

Grade 3

• Connective Tissue Disorder with secondary end-organ failure (renal, cardiac, central nervous system)

Immunological System

Acquired Immunodeficiency Syndrome (AIDS)

Grade 1
• Asymptomatic HIV+ patient
• Human Immunodeficiency virus (HIV) positive without history of AIDS defining illness, however, positive for the flu-like syndrome of early infection
• CD4 - T-lymphocyte count ⇒ CD 4+ > 200 / µl

Grade 2
• HIV + with history of defining illness which is an opportunistic infection in addition to the primary HIV infection (See list below.)
• CD 4+ < 200 / µl

Grade 3
• Fulminant AIDS with Mycobacterium Avium Intracellulare (MAI), or Pneumocystis Carinii Pneumoniae (PCP)

Note: AIDS defining illness:
1. Viral infections such as: Cytomegalovirus (CMV), Creutzfeld-Jakob (CJ) virus, Herpes simplex virus, etc.
2. Bacterial infections: Salmonella, Syphilis, bacterial pneumonias (strep, HIB)
3. Mycobacterial infections: Mycobacterium Avium-Intracellulare (MAI), Tuberculosis
4. Fungal infection: Candidiasis (oral, esophageal and vaginal infections), Cryptococcous neoformans(CNS disease), Histoplasma capsulatum (disseminated disease and septicemia), Coccidioides immitis (extensive pulmonary disease)
5. Pneumocystis Carinii Pneumonia (PCP)
6. Protozoal infection: Toxoplasmosis (causes CNS lesions with encephalopathy), Cryptosporidium (causes enteric infections)
7. Neoplasms: non-Hodgkin's lymphoma (NHL), Kaposi's sarcoma (KS).

Mini Scenario: A 32-year-old male has AIDS, pneumocystsis carinii pneumonia, and COPD. A lymph node biopsy is performed and a diagnosis of Kaposi's Sarcoma is made. KS, therefore, is the index cancer. In this situation, AIDS, pneumocytsis carinii pneumonia, and KS are all directly related to each other, so AIDS and pneumocytsis carinii should not be considered a comorbid ailment for the patient with Kaposi's Sarcoma.

Malignancy

THE INDEX CANCER SHOULD NEVER BE CODED AS A COMORBIDITY.

In the event that there are multiple primaries (i.e., synchronous or metachronous), the primary being abstracted would be the index primary, and the other primary(s) would be a comorbidity of the index primary. To determine whether multiple primaries exist, refer to the table at the end of this section adopted from the Registry Operations and Data Standards manual.

Solid Tumor

Grade 1
- Any controlled solid tumor without documented metastases, but initially diagnosed and treated greater than five years ago

Grade 2
- Any controlled solid tumor without documented metastases, but initially diagnosed and treated within the last 5 years

Grade 3
- Uncontrolled cancer (i.e., previous treatment without complete response. Patient with persistence or recurrence)
- Newly diagnosed but not yet treated (note: this includes tumors diagnosed anytime during initial treatment of the primary)
- Metastatic solid tumor

Mini Scenario 1: A female had chondrosarcoma with a finger amputation four years prior to the date of diagnosis of the index cancer. The sarcoma is controlled at the date of diagnosis. Therefore, code as a **2** for **controlled solid tumor**.

Mini Scenario 2: A male was diagnosed with prostate cancer in September of 1999. He had two previous primaries, rectal and oral cavity, in 1995 and 1996. Both the rectal and oral cavity malignancies are controlled at the date of prostate cancer diagnosis. This man would receive a **2** for **controlled solid tumor** even though he had two previous primaries within the past five years.

Mini Scenario 3: A female is diagnosed with breast cancer at the same time she is diagnosed with a separate liver cancer. In this scenario, the liver cancer would be a comorbidity of the breast cancer, and the breast cancer would be a comorbidity of the liver cancer. Each case, then, would receive a **Grade of 3** for "**Newly diagnosed but not yet treated**" due to the other synchronous tumor

Note: Always exclude from coding as malignancy:

1. Basal cell carcinoma
2. Non-invasive squamous cell carcinoma of skin.
3. Cervical carcinoma in-situ (CIS)
4. Carcinoid tumor of the appendix / small intestine
5. Paget disease of bone
6. Bowen's disease
7. Barrett's esophagus
8. Insulinoma
9. Benign brain tumors (Meningioma, Craniopharyngioma)

Note: Starting with January 1, 2001 diagnoses, the new ICD-0-3 guidelines implementation has changed the following solid tumors from borderline to malignant. Thus, include these conditions as comorbid malignancy (even if occurred prior to January 1,2001):

1. Endometrial stromal sarcoma, low grade
2. Endolymphatic stromal myosis
3. Endometrial stromatosis
4. Stromal endometriosis
5. Stromal myosis, NOS
6. Papillary ependymoma
7. Papillary meningioma

Leukemia, Myeloma, and Hematopoietic Disorders

Grade 1
- History of leukemia, myeloma, or hematopoietic disorder with last therapy greater than one year ago
- Indolent leukemia, myeloma, or hematopoietic disorder not receiving therapy (e.g. chronic lymphocytic leukemia)

Grade 2
- First remission or new diagnosis of non-indolent Leukemia, Myeloma, or Hematopoietic Disorders less than one year ago
- Chronic suppressive therapy

Grade 3
- Relapse
- Disease out of control

Mini Scenario: A male is diagnosed with lung cancer at the same time he is diagnosed with leukemia. In this scenario, the lung cancer would be a comorbidity of the leukemia, and the leukemia would be a comorbidity of the lung cancer. The lung cancer case, then, would receive a **Grade of 2** for "First remission or new diagnosis less than one year ago" due to the leukemia; the leukemia would receive a **Grade of 3** for "Newly diagnosed but not yet treated" due to the lung cancer.

Note: Leukemias as defined by the American Cancer Society include acute lymphocytic leukemia, chronic lymphocytic leukemia, acute myeloid leukemia, chronic myeloid leukemia, etc. Also, starting with January 1, 2001 diagnoses, the new ICD-0-3 implementation has changed the following conditions from borderline to malignant. Thus, code the following comorbidities as a malignancy if they occurred after January 1,2001:

1. Polycythemia vera
2. Polycythemia rubra vera
3. Chronic myeloproliferative disease, NOS
4. Chronic myeloproliferative disorder
5. Myelosclerosis w/ myeloid metaplasia
6. Megakaryocytic myelosclerosis
7. Myelofibrosis w/ myeloid metaplasia
8. Idiopathic thrombocythemia
9. Essential thrombocythemia
10. Essential hemorrhagic thrombocythemia
11. Idiopathic hemorrhagic thrombocythemia
12. Refractory anemia, NOS
13. Refractory anemia w/o sideroblasts
14. Refractory anemia w/ sideroblasts
15. Refractory anemia w/ ringed sideroblasts
16. Refractory anemia w/ excess blasts
17. Refractory anemia w excess blasts in transformation
18. Myelodysplastic syndrome, NOS
19. Preleukemia
20. Preleukemic syndrome

Lymphoma

Grade 1
• History of lymphoma with last therapy greater than one year ago
• Indolent, or low-grade, lymphoma not receiving therapy

Grade 2
• First remission or new diagnosis less than one year ago
• Chronic suppressive therapy

Grade 3
• Relapse
• Disease out of control

Note: Lymphomas as defined by the American Cancer Society include Hodgkin's disease or Non-Hodgkin's lymphoma.

Mini Scenario: A female is diagnosed with ovarian cancer at the same time she is diagnosed with lymphoma. In this scenario, the ovarian cancer would be a comorbidity of the lymphoma, and the lymphoma would be a comorbidity of the ovarian cancer. The ovarian cancer case, then, would receive a **Grade of 2** for "**First remission or new diagnosis less than one year ago**" due to the lymphoma; the lymphoma would receive a **Grade of 3** for "**Newly diagnosed but not yet treated**" due to the ovarian cancer.

Determining Multiple Primaries

LESIONS	SITE(S)	HISTOLOGY	VARIABLES	PRIMARY
Single	Single	Single		Single
	Single	Mixed/multiple		Single
Single or multiple	Single	Single	Different behavior codes, in situ (2) and invasive (3)	Single
	Same as previous site	Same as previous histology	Within two months of diagnosis	Recurrence of the original primary
	Same as previous site	Same as previous histology	More than two months after diagnosis	New primary UNLESS physician states it is metastatic.<br>**Exceptions**: Basal, Squamous, basosquamous cell carcinoma of the skin, bladder, Kaposi's sarcoma, adenocarcinoma of prostate.
Multiple	Single	Single	Simultaneous	Single
	Multiple	Single	Simultaneous	Multiple UNLESS physician states it is metastatic.<br>**Exceptions**: Ovaries (simultaneous bilateral), retinoblastoma, and Wilms' tumor are single primaries.
	Single	Mixed/multiple	Simultaneous	Single
	Single	Multiple (Each tumor has a different histology.)	Simultaneous	Multiple<br>**Exceptions**: Breast (lobular and ductal; bladder transitional and papillary).
	Multiple	Multiple	Simultaneous	Multiple

Substance Abuse

Alcohol

Grade 1

• History of alcohol abuse with social, behavioral, or medical complications, but not presently drinking

Grade 2

• Active alcohol abuse with social, behavioral, or medical complications, including: single episode of delirium tremens or alcohol-related seizure, one or more episodes of hospitalization for alcohol-associated ailments, significant behavioral problems caused by drinking ("drinking problem"), or nutritionally caused cachexia or anemia due to patient's drinking.

Grade 3

• More than one episode of delirium tremens (DT) or alcoholic seizure and currently drinking

Illicit Drugs

Grade 1

• History of substance abuse with social, behavioral, or medical complications, but not presently using

Grade 2

• Active substance abuse with social, behavioral, or medical complications

Grade 3

• Acute withdrawal syndrome

Note: Classify an individual as a current alcohol/drug abuser if they quit within six months of the date of diagnosis.

Mini Scenario: A woman with social, medical, or behavioral complications that quit drinking four months prior to the date of diagnosis would be coded as a **2** for **current drinker** with other problems.

Note: If the medical record mentions the substance, but does not suggest any associated social, behavioral, or medical complications, do not code it as Substance Abuse.

Mini Scenario: A man drinks a 12-pack of beer per day but no additional details are indicated in connection with this habit. In this case, he would receive a **0** for Substance Abuse.

Body Weight

Obesity

The influence of excess body weight on the risk of death was characterized by Calle et al (Calle EE, Rodriguez C, Walker-Thurmond K, Thun MJ: Overweight, obesity, and mortality from cancer in a prospectively studied cohort of US adults. *New England Journal of Medicine.* 2003;348:1625-1638.) Please refer to the following charts for Body Mass Index calculation. Find patient's appropriate height and weight in metric or English scale on the appropriate chart. If the point falls below the line, the patient is considered morbidly obese and should receive a comorbidity grade of 2. Do not code as morbid obesity based only on statements from the chart. Actual calculation of BMI based on height and weight is required.

Grade 1
• Not applicable

Grade 2
• Morbid Obesity (BMI $\geq$ 38)

Grade 3
• Not applicable

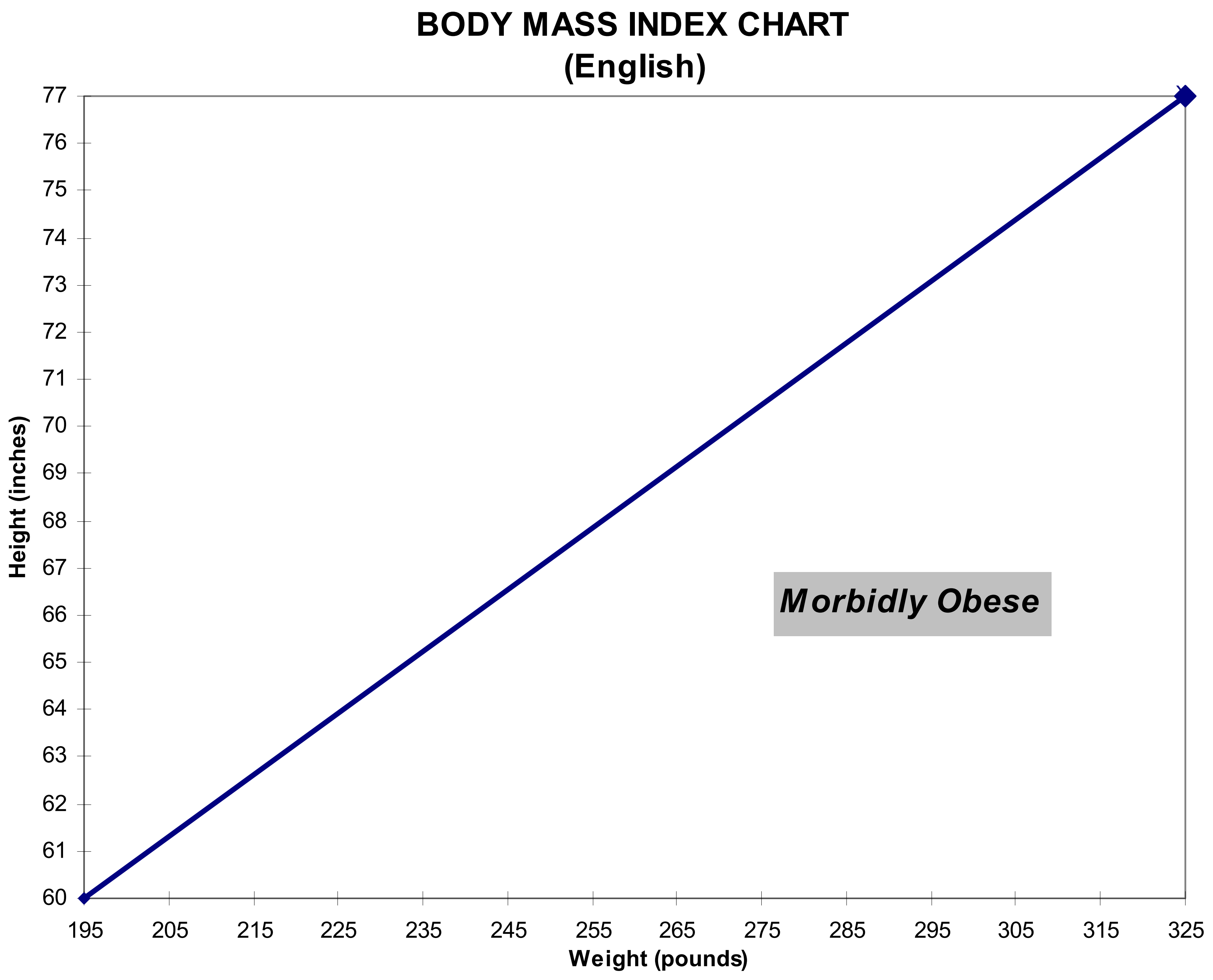
BODY MASS INDEX CHART
(English)
77
76
75
74
73
72
71
70
69
68
67
66
65
64
63
62
61
60
Height (inches)
Morbidly Obese
195
205
215
225
235
245
255
265
275
285
295
305
315
325
Weight (pounds)

BODY MASS INDEX CHART
(Metric)

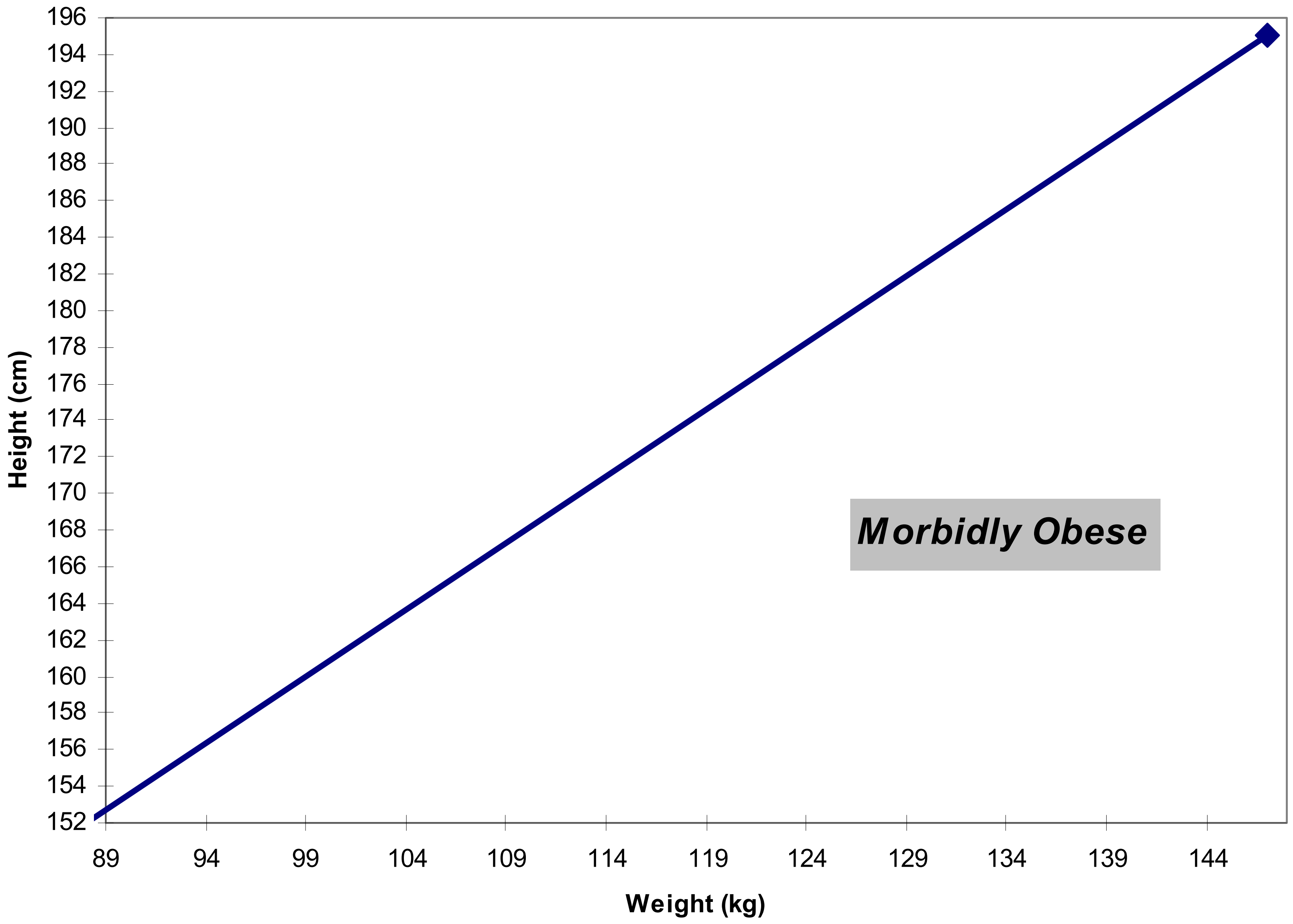

SCENARIOS

In this section, five clinical scenarios drawn from the Barnes-Jewish Hospital Oncology Data Services experience are presented to help the reader test their knowledge of comorbidity.

SCENARIO #1

DATE: September 3, 1998

CHIEF COMPLAINT: shortness of breath, dysphagia and fatigue.

HISTORY OF PRESENT ILLNESS: 54 year old male construction worker referred because of a 3 month history of shortness of breath, difficulty swallowing, and fatigue. He says that in the past 2 months he has lost approximately 25 pounds, and is constantly tired and out of breath, independent of the activity performed.
He denies hemoptysis, hoarseness, otalgia, nausea, vomiting, or neck pain.

PAST MEDICAL HISTORY:
Surgical History: Tonsillectomy and Adenoidectomy in childhood. Appendectomy many years ago.
Medical History:

Hypertension controlled with diuretic and an ACE inhibitor.
Insulin dependent Diabetes Mellitus poorly controlled.
Diagnosis of bipolar disorder currently on lithium.
+ Deep Vein Thrombosis patient currently on Coumadin.
Denies Tuberculosis, Myocardial Infarction, Heart Disease.

Medications: Lasix, Enalapril, Coumadin, Insulin, Lithium.

SOCIAL HISTORY: Smokes 2 ½ packs per day since age thirteen. Drinks 2 beers a day.
FAMILY HISTORY: Father died of heart attack at age 65.
PHYSICAL EXAM: 54 year old male with shortness of breath.

Extraocular movement: full
Ears: neg: Nose: neg.
Oral cavity/Oropharynx: neg
Nasopharynx: neg
Hypopharynx/Larynx : Large mass in the right epiglottis extending downwards making it hard to visualize the true vocal cords.
Neck: 3 cm ipsilateral neck mass, firm and fixed.
Lungs: Soft crackles bilateral
Heart : normal sinus rhythm, no murmurs.
Abdomen: Soft and non tender.
Neuro: Normal
Extremities: Left leg slightly edematous, pedal pulse diminished.
Review of symptoms: unremarkable

IMPRESSION : T4N1 Stage IVA Supraglottic Ca

Chest x-ray and labs pending.
Laryngoscopy/Biopsy scheduled
Computerized tomography scheduled.

PRIMARY DIAGNOSIS: Supraglottic squamous cell carcinoma

Date ____________________

Coder's Initials ____________

Oncology Center __________

Accession # _Scenario #1_

Adult Comorbidity Evaluation-27

Identify the important medical comorbidities and grade severity using the index. Overall Comorbidity Score is defined according to the highest ranked single ailment, except in the case where two or more Grade 2 ailments occur in different organ systems. In this situation, the overall comorbidity score should be designated Grade 3.

Cogent comorbid ailment	Grade 3 Severe Decompensation	Grade 2 Moderate Decompensation	Grade 1 Mild Decompensation
**Cardiovascular System**			
Myocardial Infarct	☐ MI ≤ 6 months	☐ MI > 6 months ago	☐ MI by ECG only, age undetermined
Angina / Coronary Artery Disease	☐ Unstable angina	☐ Chronic exertional angina<br>☐ Recent (≤ 6 months) CABG or PTCA<br>☐ Recent (≤ 6 months) coronary stent	☐ ECG or stress test evidence or catheterization evidence of coronary disease without symptoms<br>☐ Angina pectoris not requiring hospitalization<br>☐ CABG or PTCA (>6 mos.)<br>☐ Coronary stent (>6 mos.)
Congestive Heart Failure	☐ Hospitalized for CHF within past 6 months<br>☐ Ejection fraction < 20%	☐ Hospitalized for CHF >6 months prior<br>☐ CHF with dyspnea which limits activities	☐ CHF with dyspnea which has responded to treatment<br>☐ Exertional dyspnea<br>☐PND
Arrhythmias	☐ Ventricular arrhythmia ≤ 6 months	☐ Ventricular arrhythmia > 6 months ago<br>☐ Chronic atrial fibrillation or flutter<br>☐ Pacemaker	☐ Sick Sinus Syndrome
Hypertension	☐ DBP≥130 mm Hg<br>☐ Severe malignant papilledema or other eye changes<br>☐ Encephalopathy	☐ DBP 115-129 mm Hg<br>☐ DBP 90-114 mm Hg while taking antihypertensive medications<br>☐ Secondary cardiovascular symptoms: vertigo, epistaxis, headaches	☐ DBP 90-114 mm Hg while <u>not</u> taking antihypertensive medications<br>■ DBP <90 mm Hg while taking antihypertensive medications<br>☐ Hypertension NOS
Venous Disease	☐ Recent PE (≤ 6 mos.)<br>☐ Use of venous filter for PE's	■ DVT controlled with Coumadin or heparin<br>☐ Old PE > 6mos	☐ Old DVT no longer treated with Coumadin or Heparin
Peripheral Arterial Disease	☐ Bypass or amputation for gangrene or arterial insufficiency < 6 months ago<br>☐ Untreated thoracic or abdominal aneurysm (≥6 cm)	☐ Bypass or amputation for gangrene or arterial insufficiency > 6 months ago<br>☐ Chronic insufficiency	☐ Intermittent claudication<br>☐ Untreated thoracic or abdominal aneurysm (< 6 cm)<br>☐ s/p abdominal or thoracic aortic aneurysm repair
**Respiratory System**			
	☐ Marked pulmonary insufficiency<br>☐ Restrictive Lung Disease or COPD with dyspnea at rest despite treatment<br>☐ Chronic supplemental O_2<br>☐ CO_2 retention (pCO_2 > 50 torr)<br>☐ Baseline pO_2 < 50 torr<br>☐ FEV1 (< 50%)	☐ Restrictive Lung Disease or COPD (chronic bronchitis, emphysema, or asthma) with dyspnea which limits activities<br>☐ FEV1 (51%-65%)	☐ Restrictive Lung Disease or COPD (chronic bronchitis, emphysema, or asthma) with dyspnea which has responded to treatment<br>☐ FEV1 (66%-80%)
**Gastrointestinal System**			
Hepatic	☐ Portal hypertension and/or esophageal bleeding ≤ 6 mos. (Encephalopathy, Ascites, Jaundice with Total Bilirubin > 2)	☐ Chronic hepatitis, cirrhosis, portal hypertension with moderate symptoms "compensated hepatic failure"	☐ Chronic hepatitis or cirrhosis without portal hypertension<br>☐ Chronic liver disease manifested on biopsy or persistently elevated bilirubin (>3 mg/dl)
Stomach / Intestine	☐ Recent ulcers (≤ 6 months ago) requiring blood transfusion	☐ Ulcers requiring surgery or transfusion > 6 months ago	☐ Diagnosis of ulcers treated with meds<br>☐ Chronic malabsorption syndrome<br>☐ Inflammatory bowel disease (IBD) on meds or h/o with complications and/or surgery
Pancreas	☐ Acute or chronic pancreatitis with major complications (phlegmon, abscess, or pseudocyst)	☐ Uncomplicated acute pancreatitis<br>☐ Chronic pancreatitis with minor complications (malabsorption, impaired glucose tolerance, or GI bleeding)	☐ Chronic pancreatitis w/o complications

Cogent comorbid ailment	Grade 3<br>Severe Decompensation	Grade 2<br>Moderate Decompensation	Grade 1<br>Mild Decompensation
**Renal System**			
End-stage renal disease	☐ Creatinine > 3 mg% with multi-organ failure, shock, or sepsis<br>☐ Acute dialysis	☐ Chronic Renal Insufficiency with creatinine >3 mg%<br>☐ Chronic dialysis	☐ Chronic Renal Insufficiency with creatinine 2-3 mg%.
**Endocrine System**	**(Code the comorbid ailments with the (*) in both the Endocrine system and other organ systems if applicable)**		
Diabetes Mellitus	☐ Hospitalization ≤ 6 months for DKA<br>☐ Diabetes causing end-organ failure<br>☐ retinopathy<br>☐ neuropathy<br>☐ nephropathy*<br>☐ coronary disease*<br>☐ peripheral arterial disease*	■ IDDM without complications<br>☐ Poorly controlled AODM with oral agents	☐ AODM controlled by oral agents only
**Neurological System**			
Stroke	☐ Acute stroke with coma or significant neurologic deficit	☐ Old stroke with neurologic residua	☐ Stroke with no residual<br>☐ Past or recent TIA
Dementia	☐ Severe dementia requiring full support for activities of daily living	☐ Moderate dementia (not completely self-sufficient, needs supervising)	☐ Mild dementia (can take care of self)
Paralysis	☐ Paraplegia or hemiplegia requiring full support for activities of daily living	☐ Paraplegia or hemiplegia requiring wheelchair, but able to do some self care	☐ Paraplegia or hemiplegia but ambulatory and providing most of self care
Neuromuscular	☐ MS, Parkinson's or other chronic neuromuscular disorder and requiring full support for activities of daily living	☐ MS, Parkinson's, or other chronic neuromuscular disorder, but able to do some self care	☐ MS, Parkinson's, or other chronic neuromuscular disorder, but ambulatory and providing most of self care
**Psychiatric**			
	☐ Recent suicidal attempt<br>☐ Active schizophrenia	☐ Depression or bipolar disorder uncontrolled<br>☐ Schizophrenia controlled with medication	■ Depression or bipolar disorder controlled w/ medication
**Rheumatologic**	**(Including Rheumatoid Arthritis, Systemic Lupus, Mixed Connective Tissue Disorder, Polymyositis, Rheumatic Polymyositis)**		
	☐ Connective Tissue Disorder with secondary end-organ failure (renal, cardiac, CNS)	☐ Connective Tissue Disorder on steroids or immunosuppressant medications	☐ Connective Tissue Disorder on NSAIDS or no treatment
**Immunological System**			
AIDS	☐ Fulminant AIDS w/KS, MAI, PCP (AIDS defining illness)	☐ HIV+ with h/o defining illness. $CD4^+ < 200/\mu L$	☐ Asymptomatic HIV+ patient.<br>☐ HIV^+ w/o h/o AIDS defining illness. $CD4^+ > 200\mu L$
**Malignancy**	**(Excluding Cutaneous Basal Cell Ca., Cutaneous SCCA, Carcinoma in-situ, and Intraepithelial Neoplasm)**		
Solid Tumor including melanoma	☐ Uncontrolled cancer<br>☐ Newly diagnosed but not yet treated<br>☐ Metastatic solid tumor	☐ Any controlled solid tumor without documented metastases, but initially diagnosed and treated within the last 5 years	☐ Any controlled solid tumor without documented metastases, but initially diagnosed and treated > 5 years ago
Leukemia	☐ Relapse	☐ 1st remission or new dx <1yr<br>☐ Chronic suppressive therapy	☐ H/o leukemia with last Rx > 1 yr prior
Lymphoma including Myeloma	☐ Relapse	☐ 1st remission or new dx <1yr<br>☐ Chronic suppressive therapy	☐ H/o lymphoma w/ last Rx >1 yr prior
**Substance Abuse**			
Alcohol	☐ Delirium tremens	☐ Active alcohol abuse with social, behavioral, or medical complications	☐ H/o alcohol abuse but not presently drinking
Illicit Drugs	☐ Acute Withdrawal Syndrome	☐ Active substance abuse with social, behavioral, or medical complications	☐ H/o substance abuse but not presently using
**Body Weight**			
Obesity		☐ Morbid (i.e., BMI≥38)	

OVERALL COMORBIDITY SCORE 3

SCENARIO #2

DATE: February 27, 1999

CHIEF COMPLAINT: Asymptomatic patient with positive chest x-ray, referred by his primary care physician to this office for further management and treatment.

HISTORY OF PRESENT ILLNESS: 45 year old male, works for the post-office. He presented 6 months ago with weakness in his arms and legs, shortness of breath and was hospitalized and treated for Eaton-Lambert syndrome. He comes to the office referred by his primary care physician after chest x-ray showed a suspicious lung mass. He denies shortness of breath, hemoptysis, dyspnea, weight loss, anorexia, fever, or chills. He reports having a constant cough for the past two months that has been treated with antibiotics without relief.

PAST MEDICAL HISTORY:

Surgical History: Tonsillectomy and Adenoidectomy as a child.
Motor vehicle accident- multiple fractures repaired (12 years ago)

Medical History: Eaton-Lambert Syndrome diagnosed 6 months ago, patient was hospitalized and treated with plasmapheresis and immunotherapy. His primary care physician frequently sees him for follow-up on his condition.

Medications: None

SOCIAL HISTORY: Patient is a heavy smoker since age 13. Smokes approximately 2 packs per day. Drinks a six pack a day and 1-2 highballs on weekends.

FAMILY HISTORY: Father died of oral cavity cancer at age 65.

PHYSICAL EXAM : 45 year old male. BP 120/85, Temperature 37.5 C, Pulse 88.

Extraocular movement: full

Head, eyes, ears, nose, and throat: Negative

Heart: normal sinus rhythm, no murmurs.

Abdomen: Soft, non tender.

Neurologic: Deep tendon reflexes absent, proximal weakness in legs, arms and pelvic girdle remain. Cranial nerves exam - Negative

Lungs: Slight wheezing heard over right lung otherwise exam is normal.

DIAGNOSTIC TESTS :

Chest x-ray: Solitary pulmonary nodule.

IMPRESSION : Rule out **Carcinoma of the Lung**.
Bronchoscopy scheduled
CTR scheduled.

PRIMARY DIAGNOSIS: Carcinoma of the lung

Date ____________________

Coder's Initials ____________

Oncology Center __________

Accession # Scenario #2

Adult Comorbidity Evaluation-27

Identify the important medical comorbidities and grade severity using the index. Overall Comorbidity Score is defined according to the highest ranked single ailment, except in the case where two or more Grade 2 ailments occur in different organ systems. In this situation, the overall comorbidity score should be designated Grade 3.

Cogent comorbid ailment	Grade 3<br>Severe Decompensation	Grade 2<br>Moderate Decompensation	Grade 1<br>Mild Decompensation
**Cardiovascular System**			
Myocardial Infarct	☐ MI ≤ 6 months	☐ MI > 6 months ago	☐ MI by ECG only, age undetermined
Angina / Coronary Artery Disease	☐ Unstable angina	☐ Chronic exertional angina<br>☐ Recent (≤ 6 months) CABG or PTCA<br>☐ Recent (≤ 6 months) coronary stent	☐ ECG or stress test evidence or catheterization evidence of coronary disease without symptoms<br>☐ Angina pectoris not requiring hospitalization<br>☐ CABG or PTCA (>6 mos.)<br>☐ Coronary stent (>6 mos.)
Congestive Heart Failure	☐ Hospitalized for CHF within past 6 months<br>☐ Ejection fraction < 20%	☐ Hospitalized for CHF >6 months prior<br>☐ CHF with dyspnea which limits activities	☐ CHF with dyspnea which has responded to treatment<br>☐ Exertional dyspnea<br>☐PND
Arrhythmias	☐ Ventricular arrhythmia ≤ 6 months	☐ Ventricular arrhythmia > 6 months ago<br>☐ Chronic atrial fibrillation or flutter<br>☐ Pacemaker	☐ Sick Sinus Syndrome
Hypertension	☐ DBP≥130 mm Hg<br>☐ Severe malignant papilledema or other eye changes<br>☐ Encephalopathy	☐ DBP 115-129 mm Hg<br>☐ DBP 90-114 mm Hg while taking antihypertensive medications<br>☐ Secondary cardiovascular symptoms: vertigo, epistaxis, headaches	☐ DBP 90-114 mm Hg while <u>not</u> taking antihypertensive medications<br>☐ DBP <90 mm Hg while taking antihypertensive medications<br>☐ Hypertension NOS
Venous Disease	☐ Recent PE (≤ 6 mos.)<br>☐ Use of venous filter for PE's	☐ DVT controlled with Coumadin or heparin<br>☐ Old PE > 6mos	☐ Old DVT no longer treated with Coumadin or Heparin
Peripheral Arterial Disease	☐ Bypass or amputation for gangrene or arterial insufficiency < 6 months ago<br>☐ Untreated thoracic or abdominal aneurysm (≥6 cm)	☐ Bypass or amputation for gangrene or arterial insufficiency > 6 months ago<br>☐ Chronic insufficiency	☐ Intermittent claudication<br>☐ Untreated thoracic or abdominal aneurysm (< 6 cm)<br>☐ s/p abdominal or thoracic aortic aneurysm repair
**Respiratory System**			
	☐ Marked pulmonary insufficiency<br>☐ Restrictive Lung Disease or COPD with dyspnea at rest despite treatment<br>☐ Chronic supplemental O_2<br>☐ CO_2 retention (pCO_2 > 50 torr)<br>☐ Baseline pO_2 < 50 torr<br>☐ FEV1 (< 50%)	☐ Restrictive Lung Disease or COPD (chronic bronchitis, emphysema, or asthma) with dyspnea which limits activities<br>☐ FEV1 (51%-65%)	☐ Restrictive Lung Disease or COPD (chronic bronchitis, emphysema, or asthma) with dyspnea which has responded to treatment<br>☐ FEV1 (66%-80%)
**Gastrointestinal System**			
Hepatic	☐ Portal hypertension and/or esophageal bleeding ≤ 6 mos. (Encephalopathy, Ascites, Jaundice with Total Bilirubin > 2)	☐ Chronic hepatitis, cirrhosis, portal hypertension with moderate symptoms "compensated hepatic failure"	☐ Chronic hepatitis or cirrhosis without portal hypertension<br>☐ Chronic liver disease manifested on biopsy or persistently elevated bilirubin (>3 mg/dl)
Stomach / Intestine	☐ Recent ulcers (≤ 6 months ago) requiring blood transfusion	☐ Ulcers requiring surgery or transfusion > 6 months ago	☐ Diagnosis of ulcers treated with meds<br>☐ Chronic malabsorption syndrome<br>☐ Inflammatory bowel disease (IBD) on meds or h/o with complications and/or surgery
Pancreas	☐ Acute or chronic pancreatitis with major complications (phlegmon, abscess, or pseudocyst)	☐ Uncomplicated acute pancreatitis<br>☐ Chronic pancreatitis with minor complications (malabsorption, impaired glucose tolerance, or GI bleeding)	☐ Chronic pancreatitis w/o complications

Cogent comorbid ailment	Grade 3 Severe Decompensation	Grade 2 Moderate Decompensation	Grade 1 Mild Decompensation
**Renal System**			
End-stage renal disease	☐ Creatinine > 3 mg% with multi-organ failure, shock, or sepsis<br>☐ Acute dialysis	☐ Chronic Renal Insufficiency with creatinine >3 mg%<br>☐ Chronic dialysis	☐ Chronic Renal Insufficiency with creatinine 2-3 mg%.
**Endocrine System**	**(Code the comorbid ailments with the (*) in both the Endocrine system and other organ systems if applicable)**		
Diabetes Mellitus	☐ Hospitalization ≤ 6 months for DKA<br>☐ Diabetes causing end-organ failure<br>☐ retinopathy<br>☐ neuropathy<br>☐ nephropathy*<br>☐ coronary disease*<br>☐ peripheral arterial disease*	☐ IDDM without complications<br>☐ Poorly controlled AODM with oral agents	☐ AODM controlled by oral agents only
**Neurological System**			
Stroke	☐ Acute stroke with coma or significant neurologic deficit	☐ Old stroke with neurologic residua	☐ Stroke with no residual<br>☐ Past or recent TIA
Dementia	☐ Severe dementia requiring full support for activities of daily living	☐ Moderate dementia (not completely self-sufficient, needs supervising)	☐ Mild dementia (can take care of self)
Paralysis	☐ Paraplegia or hemiplegia requiring full support for activities of daily living	☐ Paraplegia or hemiplegia requiring wheelchair, but able to do some self care	☐ Paraplegia or hemiplegia but ambulatory and providing most of self care
Neuromuscular	☐ MS, Parkinson's or other chronic neuromuscular disorder and requiring full support for activities of daily living	☐ MS, Parkinson's, or other chronic neuromuscular disorder, but able to do some self care	☐ MS, Parkinson's, or other chronic neuromuscular disorder, but ambulatory and providing most of self care
**Psychiatric**			
	☐ Recent suicidal attempt<br>☐ Active schizophrenia	☐ Depression or bipolar disorder uncontrolled<br>☐ Schizophrenia controlled with medication	☐ Depression or bipolar disorder controlled w/ medication
**Rheumatologic**	**(Including Rheumatoid Arthritis, Systemic Lupus, Mixed Connective Tissue Disorder, Polymyositis, Rheumatic Polymyositis)**		
	☐ Connective Tissue Disorder with secondary end-organ failure (renal, cardiac, CNS)	☐ Connective Tissue Disorder on steroids or immunosuppressant medications	☐ Connective Tissue Disorder on NSAIDS or no treatment
**Immunological System**			
AIDS	☐ Fulminant AIDS w/KS, MAI, PCP (AIDS defining illness)	☐ HIV+ with h/o defining illness. $CD4^+ < 200/\mu L$	☐ Asymptomatic HIV+ patient.<br>☐ HIV^+ w/o h/o AIDS defining illness. $CD4^+ > 200\mu L$
**Malignancy**	**(Excluding Cutaneous Basal Cell Ca., Cutaneous SCCA, Carcinoma in-situ, and Intraepithelial Neoplasm)**		
Solid Tumor including melanoma	☐ Uncontrolled cancer<br>☐ Newly diagnosed but not yet treated<br>☐ Metastatic solid tumor	☐ Any controlled solid tumor without documented metastases, but initially diagnosed and treated within the last 5 years	☐ Any controlled solid tumor without documented metastases, but initially diagnosed and treated > 5 years ago
Leukemia	☐ Relapse	☐ 1st remission or new dx <1yr<br>☐ Chronic suppressive therapy	☐ H/o leukemia with last Rx > 1 yr prior
Lymphoma including Myeloma	☐ Relapse	☐ 1st remission or new dx <1yr<br>☐ Chronic suppressive therapy	☐ H/o lymphoma w/ last Rx >1 yr prior
**Substance Abuse**			
Alcohol	☐ Delirium tremens	☐ Active alcohol abuse with social, behavioral, or medical complications	☐ H/o alcohol abuse but not presently drinking
Illicit Drugs	☐ Acute Withdrawal Syndrome	☐ Active substance abuse with social, behavioral, or medical complications	☐ H/o substance abuse but not presently using
**Body Weight**			
Obesity		☐ Morbid (i.e., BMI≥38)	

OVERALL COMORBIDITY SCORE 0

SCENARIO # 3

DATE: December 4, 1998

CHIEF COMPLAINT: 48 year old male complains of increasing abdominal pain.

HISTORY OF PRESENT ILLNESS: 48-year old male who works for the city laying asphalt for streets. He complains that for the past four days he has been having abdominal pain that seems to increase after meals, but is not relieved with antacids. The pain is localized and of sharp quality over the left epigastric area, with some irradiation to his right. Presents today to the emergency room because the pain got unbearable and is now associated with nausea, but no vomiting. He says he has lost approximately 6 lbs in the last 5 days because he doesn't feel like eating and always feels full. Denies hematemesis, shortness of breath, fever, chills, diaphoresis, or shoulder pain.

PAST MEDICAL HISTORY:
Surgical History: Hernia repair in 1966 while in the service. Appendectomy at age 13.
Medical History: Chronic ulcers, chronic obstructive pulmonary disease
Medications: Zantac
Allergies: NKDA
SOCIAL HISTORY: Smoker of 2 packs per day for the last 30 years. Heavy drinker. Drinks approx 8-10 beers during the week and a ½ of vodka on weekends. Hospitalized in 1995 for drinking problem.
FAMILY HISTORY: Father died of lung cancer at age 65. Mother alive, mild hypertension. No siblings.
PHYSICAL EXAM : BP: 130/85, RR 30, Temperature 38 C, Pulse 98, Weight 175 lbs, Height 5'10".
Head, eyes, ears, nose, and throat: Unremarkable
Heart: normal sinus rhythm, no gallops, murmurs, or thrills.
Lungs: Ronchi over left lobe. Some wheezing over the right middle and lower lobes.
Abdomen. Left lower quadrant of abdomen scar consistent with hernia repair. + BS.
Mild tenderness to palpation over entire epigastric area, but no guarding. Gross hepatomegaly 4+ with nodular consistency to palpation and which seems to expand to the left epigastric quadrant. No signs of ascites.
Neuro: Cranial nerves 2-12 Intact.
LABORATORY RESULTS:
Chest x-ray: Large left hilar mass which seems to extend to the mediastinum. Right lower lobe appears congested. Chronic obstructive pulmonary disease changes are present.
Abdominal CT scan: Hepatomegaly. Multiple palpable masses throughout the liver consistent with liver mets.

IMPRESSION : Lung Carcinoma with liver metastasis
PRIMARY DIAGNOSIS: Stage IV Lung carcinoma

Date ___________________

Coder's Initials ____________

Oncology Center __________

Accession # _Scenario #3__

Adult Comorbidity Evaluation-27

Identify the important medical comorbidities and grade severity using the index.
Overall Comorbidity Score is defined according to the highest ranked single ailment, except in the case where two or more Grade 2 ailments occur in different organ systems. In this situation, the overall comorbidity score should be designated Grade 3.

Cogent comorbid ailment	Grade 3<br>Severe Decompensation	Grade 2<br>Moderate Decompensation	Grade 1<br>Mild Decompensation
**Cardiovascular System**			
Myocardial Infarct	☐ MI ≤ 6 months	☐ MI > 6 months ago	☐ MI by ECG only, age undetermined
Angina / Coronary Artery Disease	☐ Unstable angina	☐ Chronic exertional angina<br>☐ Recent (≤ 6 months) CABG or PTCA<br>☐ Recent (≤ 6 months) coronary stent	☐ ECG or stress test evidence or catheterization evidence of coronary disease without symptoms<br>☐ Angina pectoris not requiring hospitalization<br>☐ CABG or PTCA (>6 mos.)<br>☐ Coronary stent (>6 mos.)
Congestive Heart Failure	☐ Hospitalized for CHF within past 6 months<br>☐ Ejection fraction < 20%	☐ Hospitalized for CHF >6 months prior<br>☐ CHF with dyspnea which limits activities	☐ CHF with dyspnea which has responded to treatment<br>☐ Exertional dyspnea<br>☐PND
Arrhythmias	☐ Ventricular arrhythmia ≤ 6 months	☐ Ventricular arrhythmia > 6 months ago<br>☐ Chronic atrial fibrillation or flutter<br>☐ Pacemaker	☐ Sick Sinus Syndrome
Hypertension	☐ DBP≥130 mm Hg<br>☐ Severe malignant papilledema or other eye changes<br>☐ Encephalopathy	☐ DBP 115-129 mm Hg<br>☐ DBP 90-114 mm Hg while taking antihypertensive medications<br>☐ Secondary cardiovascular symptoms: vertigo, epistaxis, headaches	☐ DBP 90-114 mm Hg while <u>not</u> taking antihypertensive medications<br>☐ DBP <90 mm Hg while taking antihypertensive medications<br>☐ Hypertension NOS
Venous Disease	☐ Recent PE (≤ 6 mos.)<br>☐ Use of venous filter for PE's	☐ DVT controlled with Coumadin or heparin<br>☐ Old PE > 6mos	☐ Old DVT no longer treated with Coumadin or Heparin
Peripheral Arterial Disease	☐ Bypass or amputation for gangrene or arterial insufficiency < 6 months ago<br>☐ Untreated thoracic or abdominal aneurysm (≥6 cm)	☐ Bypass or amputation for gangrene or arterial insufficiency > 6 months ago<br>☐ Chronic insufficiency	☐ Intermittent claudication<br>☐ Untreated thoracic or abdominal aneurysm (< 6 cm)<br>☐ s/p abdominal or thoracic aortic aneurysm repair
**Respiratory System**			
	☐ Marked pulmonary insufficiency<br>☐ Restrictive Lung Disease or COPD with dyspnea at rest despite treatment<br>☐ Chronic supplemental O_2<br>☐ CO_2 retention (pCO_2 > 50 torr)<br>☐ Baseline pO_2 < 50 torr<br>☐ FEV1 (< 50%)	☐ Restrictive Lung Disease or COPD (chronic bronchitis, emphysema, or asthma) with dyspnea which limits activities<br>☐ FEV1 (51%-65%)	■ Restrictive Lung Disease or COPD (chronic bronchitis, emphysema, or asthma) with dyspnea which has responded to treatment<br>☐ FEV1 (66%-80%)
**Gastrointestinal System**			
Hepatic	☐ Portal hypertension and/or esophageal bleeding ≤ 6 mos. (Encephalopathy, Ascites, Jaundice with Total Bilirubin > 2)	☐ Chronic hepatitis, cirrhosis, portal hypertension with moderate symptoms "compensated hepatic failure"	☐ Chronic hepatitis or cirrhosis without portal hypertension<br>☐ Chronic liver disease manifested on biopsy or persistently elevated bilirubin (>3 mg/dl)
Stomach / Intestine	☐ Recent ulcers (≤ 6 months ago) requring blood transfusion	☐ Ulcers requiring surgery or transfusion > 6 months ago	■ Diagnosis of ulcers treated with meds<br>☐ Chronic malabsorption syndrome<br>☐ Inflammatory bowel disease (IBD) on meds or h/o with complications and/or surgery
Pancreas	☐ Acute or chronic pancreatitis with major complications (phlegmon, abscess, or pseudocyst)	☐ Uncomplicated acute pancreatitis<br>☐ Chronic pancreatitis with minor complications (malabsorption, impaired glucose tolerance, or GI bleeding)	☐ Chronic pancreatitis w/o complications

Cogent comorbid ailment	Grade 3<br>Severe Decompensation	Grade 2<br>Moderate Decompensation	Grade 1<br>Mild Decompensation
**Renal System**			
End-stage renal disease	☐ Creatinine > 3 mg% with multi-organ failure, shock, or sepsis<br>☐ Acute dialysis	☐ Chronic Renal Insufficiency with creatinine >3 mg%<br>☐ Chronic dialysis	☐ Chronic Renal Insufficiency with creatinine 2-3 mg%.
**Endocrine System**	**(Code the comorbid ailments with the (*) in both the Endocrine system and other organ systems if applicable)**		
Diabetes Mellitus	☐ Hospitalization ≤ 6 months for DKA<br>☐ Diabetes causing end-organ failure<br>☐ retinopathy<br>☐ neuropathy<br>☐ nephropathy*<br>☐ coronary disease*<br>☐ peripheral arterial disease*	☐ IDDM without complications<br>☐ Poorly controlled AODM with oral agents	☐ AODM controlled by oral agents only
**Neurological System**			
Stroke	☐ Acute stroke with coma or significant neurologic deficit	☐ Old stroke with neurologic residua	☐ Stroke with no residual<br>☐ Past or recent TIA
Dementia	☐ Severe dementia requiring full support for activities of daily living	☐ Moderate dementia (not completely self-sufficient, needs supervising)	☐ Mild dementia (can take care of self)
Paralysis	☐ Paraplegia or hemiplegia requiring full support for activities of daily living	☐ Paraplegia or hemiplegia requiring wheelchair, but able to do some self care	☐ Paraplegia or hemiplegia but ambulatory and providing most of self care
Neuromuscular	☐ MS, Parkinson's or other chronic neuromuscular disorder and requiring full support for activities of daily living	☐ MS, Parkinson's, or other chronic neuromuscular disorder, but able to do some self care	☐ MS, Parkinson's, or other chronic neuromuscular disorder, but ambulatory and providing most of self care
**Psychiatric**			
	☐ Recent suicidal attempt<br>☐ Active schizophrenia	☐ Depression or bipolar disorder uncontrolled<br>☐ Schizophrenia controlled with medication	☐ Depression or bipolar disorder controlled w/ medication
**Rheumatologic**	**(Including Rheumatoid Arthritis, Systemic Lupus, Mixed Connective Tissue Disorder, Polymyositis, Rheumatic Polymyositis)**		
	☐ Connective Tissue Disorder with secondary end-organ failure (renal, cardiac, CNS)	☐ Connective Tissue Disorder on steroids or immunosuppressant medications	☐ Connective Tissue Disorder on NSAIDS or no treatment
**Immunological System**			
AIDS	☐ Fulminant AIDS w/KS, MAI, PCP (AIDS defining illness)	☐ HIV+ with h/o defining illness. $CD4^+ < 200/\mu L$	☐ Asymptomatic HIV+ patient.<br>☐ HIV^+ w/o h/o AIDS defining illness. $CD4^+ > 200\mu L$
**Malignancy**	**(Excluding Cutaneous Basal Cell Ca., Cutaneous SCCA, Carcinoma in-situ, and Intraepithelial Neoplasm)**		
Solid Tumor including melanoma	☐ Uncontrolled cancer<br>☐ Newly diagnosed but not yet treated<br>☐ Metastatic solid tumor	☐ Any controlled solid tumor without documented metastases, but initially diagnosed and treated within the last 5 years	☐ Any controlled solid tumor without documented metastases, but initially diagnosed and treated > 5 years ago
Leukemia	☐ Relapse	☐ 1st remission or new dx <1yr<br>☐ Chronic suppressive therapy	☐ H/o leukemia with last Rx > 1 yr prior
Lymphoma including Myeloma	☐ Relapse	☐ 1st remission or new dx <1yr<br>☐ Chronic suppressive therapy	☐ H/o lymphoma w/ last Rx >1 yr prior
**Substance Abuse**			
Alcohol	☐ Delirium tremens	■ Active alcohol abuse with social, behavioral, or medical complications	☐ H/o alcohol abuse but not presently drinking
Illicit Drugs	☐ Acute Withdrawal Syndrome	☐ Active substance abuse with social, behavioral, or medical complications	☐ H/o substance abuse but not presently using
**Body Weight**			
Obesity		☐ Morbid (i.e., BMI≥38)	

OVERALL COMORBIDITY SCORE <u>2</u>

SCENARIO # 4

DATE: January 14, 1998

CHIEF COMPLAINT: shortness of breath, weight loss

HISTORY OF PRESENT ILLNESS: 67-year old male presented to private medical doctor with 4 month history of dyspnea on exertion, 2 pillow orthopnea, 15 lb. weight loss, and no hemoptysis or cough. Denies myocardial infarction, diabetes mellitus, heart disease, peptic ulcer disease, tuberculosis, or peripheral vascular disease.

PAST MEDICAL HISTORY:
Surgical History: Hernia repair in 1983.
Complete excision of malignant melanoma in October 1993.
Medical History: hypertension, atrial fibrillation, cerebrovascular accident 1991
Medications: VasoTec, Coumadin
Allergies: no known allergies

SOCIAL HISTORY: Married, retired, former factory worker. Smoker of 1 pack per day for 45 years. Quit in 1991. Socially drinks. Denies intravenous drug abuse.

FAMILY HISTORY: Mother died at the age of 72 from breast cancer. Father died at the age of 69 with cerebrovascular accident. Brother has prostate cancer.

PHYSICAL EXAM : 67 year old male with shortness of breath
Extraocular movement: full
Head, eyes, ears, nose, and throat: neg
Lungs: decreased breath sounds R upper lobe
Abdomen: neg
Heart: normal sinus rhythm, no murmurs
Neuro: neg
Extremities: Intact

LABS: Chest CT and Chest x-ray, 3.2 x 2.7 x 2.3 cm mass right upper lobe mass consistent with bronchiogenic carcinoma, several enlarged peripheral lymph nodes (largest 2.3 cm), no mediastinal adenopathy

PATH: Fine needle aspiration of right upper lobe - poorly differentiated non-small cell carcinoma

PRIMARY DIAGNOSIS: Stage IIIA primary bronchiogenic carcinoma with **perihilar lymphadenopathy**

Date ____________________

Coder's Initials ____________

Oncology Center __________

Accession #   Scenario #4

Adult Comorbidity Evaluation-27

Identify the important medical comorbidities and grade severity using the index.
Overall Comorbidity Score is defined according to the highest ranked single ailment, except in the case where two or more Grade 2 ailments occur in different organ systems. In this situation, the overall comorbidity score should be designated Grade 3.

**Cogent comorbid ailment**	**Grade 3<br>Severe Decompensation**	**Grade 2<br>Moderate Decompensation**	**Grade 1<br>Mild Decompensation**
**Cardiovascular System**			
Myocardial Infarct	☐ MI ≤ 6 months	☐ MI > 6 months ago	☐ MI by ECG only, age undetermined
Angina / Coronary Artery Disease	☐ Unstable angina	☐ Chronic exertional angina<br>☐ Recent (≤ 6 months) CABG or PTCA<br>☐ Recent (≤ 6 months) coronary stent	☐ ECG or stress test evidence or catheterization evidence of coronary disease without symptoms<br>☐ Angina pectoris not requiring hospitalization<br>☐ CABG or PTCA (>6 mos.)<br>☐ Coronary stent (>6 mos.)
Congestive Heart Failure	☐ Hospitalized for CHF within past 6 months<br>☐ Ejection fraction < 20%	☐ Hospitalized for CHF >6 months prior<br>☐ CHF with dyspnea which limits activities	☐ CHF with dyspnea which has responded to treatment<br>☐ Exertional dyspnea<br>☐PND
Arrhythmias	☐ Ventricular arrhythmia ≤ 6 months	☐ Ventricular arrhythmia > 6 months ago<br>■ Chronic atrial fibrillation or flutter<br>☐ Pacemaker	☐ Sick Sinus Syndrome
Hypertension	☐ DBP≥130 mm Hg<br>☐ Severe malignant papilledema or other eye changes<br>☐ Encephalopathy	☐ DBP 115-129 mm Hg<br>☐ DBP 90-114 mm Hg while taking antihypertensive medications<br>☐ Secondary cardiovascular symptoms: vertigo, epistaxis, headaches	☐ DBP 90-114 mm Hg while <u>not</u> taking antihypertensive medications<br>■ DBP <90 mm Hg while taking antihypertensive medications<br>☐ Hypertension NOS
Venous Disease	☐ Recent PE (≤ 6 mos.)<br>☐ Use of venous filter for PE's	☐ DVT controlled with Coumadin or heparin<br>☐ Old PE > 6mos	☐ Old DVT no longer treated with Coumadin or Heparin
Peripheral Arterial Disease	☐ Bypass or amputation for gangrene or arterial insufficiency < 6 months ago<br>☐ Untreated thoracic or abdominal aneurysm (≥6 cm)	☐ Bypass or amputation for gangrene or arterial insufficiency > 6 months ago<br>☐ Chronic insufficiency	☐ Intermittent claudication<br>☐ Untreated thoracic or abdominal aneurysm (< 6 cm)<br>☐ s/p abdominal or thoracic aortic aneurysm repair
**Respiratory System**			
	☐ Marked pulmonary insufficiency<br>☐ Restrictive Lung Disease or COPD with dyspnea at rest despite treatment<br>☐ Chronic supplemental O_2<br>☐ CO_2 retention (pCO_2 > 50 torr)<br>☐ Baseline pO_2 < 50 torr<br>☐ FEV1 (< 50%)	☐ Restrictive Lung Disease or COPD (chronic bronchitis, emphysema, or asthma) with dyspnea which limits activities<br>☐ FEV1 (51%-65%)	☐ Restrictive Lung Disease or COPD (chronic bronchitis, emphysema, or asthma) with dyspnea which has responded to treatment<br>☐ FEV1 (66%-80%)
**Gastrointestinal System**			
Hepatic	☐ Portal hypertension and/or esophageal bleeding ≤ 6 mos. (Encephalopathy, Ascites, Jaundice with Total Bilirubin > 2)	☐ Chronic hepatitis, cirrhosis, portal hypertension with moderate symptoms "compensated hepatic failure"	☐ Chronic hepatitis or cirrhosis without portal hypertension<br>☐ Chronic liver disease manifested on biopsy or persistently elevated bilirubin (>3 mg/dl)
Stomach / Intestine	☐ Recent ulcers (≤ 6 months ago) requiring blood transfusion	☐ Ulcers requiring surgery or transfusion > 6 months ago	☐ Diagnosis of ulcers treated with meds<br>☐ Chronic malabsorption syndrome<br>☐ Inflammatory bowel disease (IBD) on meds or h/o with complications and/or surgery
Pancreas	☐ Acute or chronic pancreatitis with major complications (phlegmon, abscess, or pseudocyst)	☐ Uncomplicated acute pancreatitis<br>☐ Chronic pancreatitis with minor complications (malabsorption, impaired glucose tolerance, or GI bleeding)	☐ Chronic pancreatitis w/o complications

Cogent comorbid ailment	Grade 3<br>Severe Decompensation	Grade 2<br>Moderate Decompensation	Grade 1<br>Mild Decompensation
**Renal System**			
End-stage renal disease	☐ Creatinine > 3 mg% with multi-organ failure, shock, or sepsis<br>☐ Acute dialysis	☐ Chronic Renal Insufficiency with creatinine >3 mg%<br>☐ Chronic dialysis	☐ Chronic Renal Insufficiency with creatinine 2-3 mg%.
**Endocrine System**	**(Code the comorbid ailments with the (*) in both the Endocrine system and other organ systems if applicable)**		
Diabetes Mellitus	☐ Hospitalization ≤ 6 months for DKA<br>☐ Diabetes causing end-organ failure<br>☐ retinopathy<br>☐ neuropathy<br>☐ nephropathy*<br>☐ coronary disease*<br>☐ peripheral arterial disease*	☐ IDDM without complications<br>☐ Poorly controlled AODM with oral agents	☐ AODM controlled by oral agents only
**Neurological System**			
Stroke	☐ Acute stroke with coma or significant neurologic deficit	☐ Old stroke with neurologic residua	■ Stroke with no residual<br>☐ Past or recent TIA
Dementia	☐ Severe dementia requiring full support for activities of daily living	☐ Moderate dementia (not completely self-sufficient, needs supervising)	☐ Mild dementia (can take care of self)
Paralysis	☐ Paraplegia or hemiplegia requiring full support for activities of daily living	☐ Paraplegia or hemiplegia requiring wheelchair, but able to do some self care	☐ Paraplegia or hemiplegia but ambulatory and providing most of self care
Neuromuscular	☐ MS, Parkinson's or other chronic neuromuscular disorder and requiring full support for activities of daily living	☐ MS, Parkinson's, or other chronic neuromuscular disorder, but able to do some self care	☐ MS, Parkinson's, or other chronic neuromuscular disorder, but ambulatory and providing most of self care
**Psychiatric**			
	☐ Recent suicidal attempt<br>☐ Active schizophrenia	☐ Depression or bipolar disorder uncontrolled<br>☐ Schizophrenia controlled with medication	☐ Depression or bipolar disorder controlled w/ medication
**Rheumatologic**	**(Including Rheumatoid Arthritis, Systemic Lupus, Mixed Connective Tissue Disorder, Polymyositis, Rheumatic Polymyositis)**		
	☐ Connective Tissue Disorder with secondary end-organ failure (renal, cardiac, CNS)	☐ Connective Tissue Disorder on steroids or immunosuppressant medications	☐ Connective Tissue Disorder on NSAIDS or no treatment
**Immunological System**			
AIDS	☐ Fulminant AIDS w/KS, MAI, PCP (AIDS defining illness)	☐ HIV+ with h/o defining illness. $CD4^+ < 200/\mu L$	☐ Asymptomatic HIV+ patient.<br>☐ HIV^+ w/o h/o AIDS defining illness. $CD4^+ > 200\mu L$
**Malignancy**	**(Excluding Cutaneous Basal Cell Ca., Cutaneous SCCA, Carcinoma in-situ, and Intraepithelial Neoplasm)**		
Solid Tumor including melanoma	☐ Uncontrolled cancer<br>☐ Newly diagnosed but not yet treated<br>☐ Metastatic solid tumor	■ Any controlled solid tumor without documented metastases, but initially diagnosed and treated within the last 5 years	☐ Any controlled solid tumor without documented metastases, but initially diagnosed and treated > 5 years ago
Leukemia	☐ Relapse	☐ 1st remission or new dx <1yr<br>☐ Chronic suppressive therapy	☐ H/o leukemia with last Rx > 1 yr prior
Lymphoma including Myeloma	☐ Relapse	☐ 1st remission or new dx <1yr<br>☐ Chronic suppressive therapy	☐ H/o lymphoma w/ last Rx >1 yr prior
**Substance Abuse**			
Alcohol	☐ Delirium tremens	☐ Active alcohol abuse with social, behavioral, or medical complications	☐ H/o alcohol abuse but not presently drinking
Illicit Drugs	☐ Acute Withdrawal Syndrome	☐ Active substance abuse with social, behavioral, or medical complications	☐ H/o substance abuse but not presently using
**Body Weight**			
Obesity		☐ Morbid (i.e., BMI≥38)	

OVERALL COMORBIDITY SCORE <u>3</u>

SCENARIO # 5

DATE: July 15, 1998

CHIEF COMPLAINT: Routine follow-up yearly mammogram

HISTORY OF PRESENT ILLNESS: 48 year old female presented for yearly mammogram. Patient was informed of findings of 2.0 cm spiculated mass in left upper outer quadrant. Patient saw surgeon who performed needle biopsy. Results showed ductal carcinoma with small couredo in-situ component. Patient present for Modified Radical Mastectomy.

PAST MEDICAL HISTORY:
Surgical History: Tonsillectomy and Adenoidectomy as child
Medical History: Depression
Medications: Elivil
Allergies: No known allergies

SOCIAL HISTORY: Patient is Gravida 4 Para 3. Patient is married and is a vice president for a large marketing corporation. She denies smoking history, social drinker, no history intravenous drug abuse.

FAMILY HISTORY: Maternal Grandmother with breast cancer, sister breast cancer, brother with hypertension and myocardial infarction. Father died age 74 of myocardial infarction. Mother died age 72 with cerebrovascular accident.

PHYSICAL EXAM : 48 year old female
Extraocular movement: full
Head, eyes, ears, nose, and throat: neg
Lungs: clear
Heart : normal sinus rhythm
Breast: No palpable abnormalities
Abdomen: neg
Neuro: neg
Extremities: intact

LABS: Chest x-ray: no evidence of disease, Mammogram: As above, Estrogen Receptor / Progesterone Receptor: pos by immunohistochemical stains

PRIMARY DIAGNOSIS: Stage I left upper outer quadrant ductal adenocarcinoma, **no palpable lymphadenopathy**

Date ___________________

Coder's Initials ____________

Oncology Center __________

Accession # _Scenario #5__

Adult Comorbidity Evaluation-27

Identify the important medical comorbidities and grade severity using the index.
Overall Comorbidity Score is defined according to the highest ranked single ailment, except in the case where two or more Grade 2 ailments occur in different organ systems. In this situation, the overall comorbidity score should be designated Grade 3.

Cogent comorbid ailment	Grade 3<br>Severe Decompensation	Grade 2<br>Moderate Decompensation	Grade 1<br>Mild Decompensation
**Cardiovascular System**			
Myocardial Infarct	☐ MI ≤ 6 months	☐ MI > 6 months ago	☐ MI by ECG only, age undetermined
Angina / Coronary Artery Disease	☐ Unstable angina	☐ Chronic exertional angina<br>☐ Recent (≤ 6 months) CABG or PTCA<br>☐ Recent (≤ 6 months) coronary stent	☐ ECG or stress test evidence or catheterization evidence of coronary disease without symptoms<br>☐ Angina pectoris not requiring hospitalization<br>☐ CABG or PTCA (>6 mos.)<br>☐ Coronary stent (>6 mos.)
Congestive Heart Failure	☐ Hospitalized for CHF within past 6 months<br>☐ Ejection fraction < 20%	☐ Hospitalized for CHF >6 months prior<br>☐ CHF with dyspnea which limits activities	☐ CHF with dyspnea which has responded to treatment<br>☐ Exertional dyspnea<br>☐PND
Arrhythmias	☐ Ventricular arrhythmia ≤ 6 months	☐ Ventricular arrhythmia > 6 months ago<br>☐ Chronic atrial fibrillation or flutter<br>☐ Pacemaker	☐ Sick Sinus Syndrome
Hypertension	☐ DBP≥130 mm Hg<br>☐ Severe malignant papilledema or other eye changes<br>☐ Encephalopathy	☐ DBP 115-129 mm Hg<br>☐ DBP 90-114 mm Hg while taking antihypertensive medications<br>☐ Secondary cardiovascular symptoms: vertigo, epistaxis, headaches	☐ DBP 90-114 mm Hg while not taking antihypertensive medications<br>☐ DBP <90 mm Hg while taking antihypertensive medications<br>☐ Hypertension NOS
Venous Disease	☐ Recent PE (≤ 6 mos.)<br>☐ Use of venous filter for PE's	☐ DVT controlled with Coumadin or heparin<br>☐ Old PE > 6mos	☐ Old DVT no longer treated with Coumadin or Heparin
Peripheral Arterial Disease	☐ Bypass or amputation for gangrene or arterial insufficiency < 6 months ago<br>☐ Untreated thoracic or abdominal aneurysm (≥6 cm)	☐ Bypass or amputation for gangrene or arterial insufficiency > 6 months ago<br>☐ Chronic insufficiency	☐ Intermittent claudication<br>☐ Untreated thoracic or abdominal aneurysm (< 6 cm)<br>☐ s/p abdominal or thoracic aortic aneurysm repair
**Respiratory System**			
	☐ Marked pulmonary insufficiency<br>☐ Restrictive Lung Disease or COPD with dyspnea at rest despite treatment<br>☐ Chronic supplemental O_2<br>☐ CO_2 retention (pCO_2 > 50 torr)<br>☐ Baseline pO_2 < 50 torr<br>☐ FEV1 (< 50%)	☐ Restrictive Lung Disease or COPD (chronic bronchitis, emphysema, or asthma) with dyspnea which limits activities<br>☐ FEV1 (51%-65%)	☐ Restrictive Lung Disease or COPD (chronic bronchitis, emphysema, or asthma) with dyspnea which has responded to treatment<br>☐ FEV1 (66%-80%)
**Gastrointestinal System**			
Hepatic	☐ Portal hypertension and/or esophageal bleeding ≤ 6 mos. (Encephalopathy, Ascites, Jaundice with Total Bilirubin > 2)	☐ Chronic hepatitis, cirrhosis, portal hypertension with moderate symptoms "compensated hepatic failure"	☐ Chronic hepatitis or cirrhosis without portal hypertension<br>☐ Chronic liver disease manifested on biopsy or persistently elevated bilirubin (>3 mg/dl)
Stomach / Intestine	☐ Recent ulcers (≤ 6 months ago) requiring blood transfusion	☐ Ulcers requiring surgery or transfusion > 6 months ago	☐ Diagnosis of ulcers treated with meds<br>☐ Chronic malabsorption syndrome<br>☐ Inflammatory bowel disease (IBD) on meds or h/o with complications and/or surgery
Pancreas	☐ Acute or chronic pancreatitis with major complications (phlegmon, abscess, or pseudocyst)	☐ Uncomplicated acute pancreatitis<br>☐ Chronic pancreatitis with minor complications (malabsorption, impaired glucose tolerance, or GI bleeding)	☐ Chronic pancreatitis w/o complications

Cogent comorbid	Grade 3	Grade 2	Grade 1

ailment	Severe Decompensation	Moderate Decompensation	Mild Decompensation
**Renal System**			
End-stage renal disease	☐ Creatinine > 3 mg% with multi-organ failure, shock, or sepsis<br>☐ Acute dialysis	☐ Chronic Renal Insufficiency with creatinine >3 mg%<br>☐ Chronic dialysis	☐ Chronic Renal Insufficiency with creatinine 2-3 mg%.
**Endocrine System**	**(Code the comorbid ailments with the (*) in both the Endocrine system and other organ systems if applicable)**		
Diabetes Mellitus	☐ Hospitalization ≤ 6 months for DKA<br>☐ Diabetes causing end-organ failure<br>☐ retinopathy<br>☐ neuropathy<br>☐ nephropathy*<br>☐ coronary disease*<br>☐ peripheral arterial disease*	☐ IDDM without complications<br>☐ Poorly controlled AODM with oral agents	☐ AODM controlled by oral agents only
**Neurological System**			
Stroke	☐ Acute stroke with coma or significant neurologic deficit	☐ Old stroke with neurologic residua	☐ Stroke with no residual<br>☐ Past or recent TIA
Dementia	☐ Severe dementia requiring full support for activities of daily living	☐ Moderate dementia (not completely self-sufficient, needs supervising)	☐ Mild dementia (can take care of self)
Paralysis	☐ Paraplegia or hemiplegia requiring full support for activities of daily living	☐ Paraplegia or hemiplegia requiring wheelchair, but able to do some self care	☐ Paraplegia or hemiplegia but ambulatory and providing most of self care
Neuromuscular	☐ MS, Parkinson's or other chronic neuromuscular disorder and requiring full support for activities of daily living	☐ MS, Parkinson's, or other chronic neuromuscular disorder, but able to do some self care	☐ MS, Parkinson's, or other chronic neuromuscular disorder, but ambulatory and providing most of self care
**Psychiatric**			
	☐ Recent suicidal attempt<br>☐ Active schizophrenia	☐ Depression or bipolar disorder uncontrolled<br>☐ Schizophrenia controlled with medication	■ Depression or bipolar disorder controlled w/ medication
**Rheumatologic**	**(Including Rheumatoid Arthritis, Systemic Lupus, Mixed Connective Tissue Disorder, Polymyositis, Rheumatic Polymyositis)**		
	☐ Connective Tissue Disorder with secondary end-organ failure (renal, cardiac, CNS)	☐ Connective Tissue Disorder on steroids or immunosuppressant medications	☐ Connective Tissue Disorder on NSAIDS or no treatment
**Immunological System**			
AIDS	☐ Fulminant AIDS w/KS, MAI, PCP (AIDS defining illness)	☐ HIV+ with h/o defining illness. $CD4^+ < 200/\mu L$	☐ Asymptomatic HIV+ patient.<br>☐ HIV^+ w/o h/o AIDS defining illness. $CD4^+ > 200\mu L$
**Malignancy**	**(Excluding Cutaneous Basal Cell Ca., Cutaneous SCCA, Carcinoma in-situ, and Intraepithelial Neoplasm)**		
Solid Tumor including melanoma	☐ Uncontrolled cancer<br>☐ Newly diagnosed but not yet treated<br>☐ Metastatic solid tumor	☐ Any controlled solid tumor without documented metastases, but initially diagnosed and treated within the last 5 years	☐ Any controlled solid tumor without documented metastases, but initially diagnosed and treated > 5 years ago
Leukemia	☐ Relapse	☐ 1st remission or new dx <1yr<br>☐ Chronic suppressive therapy	☐ H/o leukemia with last Rx > 1 yr prior
Lymphoma including Myeloma	☐ Relapse	☐ 1st remission or new dx <1yr<br>☐ Chronic suppressive therapy	☐ H/o lymphoma w/ last Rx >1 yr prior
**Substance Abuse**			
Alcohol	☐ Delirium tremens	☐ Active alcohol abuse with social, behavioral, or medical complications	☐ H/o alcohol abuse but not presently drinking
Illicit Drugs	☐ Acute Withdrawal Syndrome	☐ Active substance abuse with social, behavioral, or medical complications	☐ H/o substance abuse but not presently using
**Body Weight**			
Obesity		☐ Morbid (i.e., BMI≥38)	

OVERALL COMORBIDITY SCORE 1

INDEX OF ABBREVIATIONS OF PROCEDURES AND DISEASES

COMMON ABBREVIATIONS FOR DISEASES

Abbreviation	Disease	System
AIDS	Acquired Immune deficiency syndrome	Immune
ALL	Acute Lymphocytic Leukemia	Hematologic
AML	Acute Myelogenous Leukemia	Hematologic
ARDS	Acute Respiratory Distress syndrome	Respiratory
ATN	Acute Tubular Necrosis	Renal
ARF	Acute renal failure	Renal
AODM	Adult onset Diabetes Mellitus	Endocrine
ALS	Amyotrophic Lateral Sclerosis	Neurologic
A.S.	Aortic stenosis	Cardiovascular
ASHD	Arteriosclerotic heart disease	Cardiovascular
AS	Artherosclerosis	Cardiovascular
AI	Atrial insufficiency	Cardiovascular
APC	Atrial premature complexes	Cardiovascular
CVD	Cardiovascular disease	Cardiovascular
CVA	Cerebrovascular accident	Neurology
CLL	Chronic Lymphocytic Leukemia	Hematologic
CML	Chronic Myelogenous Leukemia	Hematologic
COLD	Chronic obstructive lung disease	Respiratory
COPD	Chronic obstructive lung disease	Respiratory
CRF	Chronic renal failure	Renal
CUC	chronic ulcerative colitis	Gastrointestinal
CHF	Congestive heart failure	Cardiovascular
CAD	Coronary artery disease	Cardiovascular
CF	Cystic Fibrosis	Gastrointestinal-Respiratory
DVT	Deep vein thrombosis	Cardiovascular
DJD	Degenerative joint disease	Musculoskeletal
DM	Dermatomyositis	Rheumatologic
DKA	Diabetic ketoacidosis	Endocrine
DIC	Disseminated intravascular coagulop	Hematologic
ESRD	End stage renal disease	Renal
GER	Gastroesophageal reflux	Gastrointestinal
HAV	Hepatitis A (virus)	Hepatic (GI)
HBV	Hepatitis B (virus)	Hepatic (GI)
HCV	Hepatitis C (virus)	Hepatic (GI)
HTN	Hypertension	Cardiovascular
HCVD	Hypertensive cardiovascular disease	Cardiovascular
IHSS	Idiopathic hypertrophic subaortic site	Cardiovascular
ITP	Idiopathic thrombocytopenia	Hematologic
IH	Infectious hepatitis	Hepatic (GI)
IBD	Inflammatory bowel disease	Gastrointestinal
IDDM	Insulin dependent Diabetes Mellitus	Endocrine
ILD	Interstitial lung disease	Respiratory
JRA	Juvenile rheumatoid arthritis	Rheumatologic
KS	Kaposi Sarcoma	
LBBB	Left bundle branch block	Cardiovascular

Abbreviation	Disease	System
M.S.	Mitral stenosis	Cardiovascular
MVP	Mitral valve prolapse	Cardiovascular
MCID	Mixed combined immunodeficiency	Immune
MCTD	Mixed connective tissue disease	Rheumatologic
MAT	Mutifocal arterial tachycardia	Cardiovascular
MPVC	Multifocal premature ventricular cont	Cardiovascular
MS	Multiple Sclerosis	Neurologic
MM	Multiple myeloma	Immune
MG	Myasthenia Gravis	Neurologic
MI	Myocardial infarction	Cardiovascular
OBS	Organic brain syndrome	Neurologic
PND	Paroxysmal Nocturnal Dyspnea	Cardiovascular
PNH	Paroxysmal Nocturnal Hemogobinur	Hematologic
PAT	Paroxysmal atrial tachycardia	Cardiovascular
PID	Pelvic inflammatory disease	Reproductive
PUD	Peptic ulcer disease	Gastrointestinal
PVD	Peripheral vascular disease	Cardiovascular
PV	Polycythemia Vera	Hematologic
PM	Polymyositis	Rheumatologic
PAC	Premature atrial contraction	Cardiovascular
PSS	Progressive systemic sclerosis	Neurologic
RA	Rheumatoid arthritis	Rheumatologic
RHD	Rheumatic heart disease	Cardiovascular
RBBB	Right bundle branch block	Cardiovascular
SCID	Severe combined immunodeficiency	Immune
SSS	Sick Sinus syndrome	Cardiovascular
SIADH	Syndrome of inappropriate ADH	Endocrine
SLE	Systemic lupus erythematous	Rheumatologic
TTP	Thrombotic thrombocytopenic purpur	Hematologic
TIA	Transient ischemic attack	Neurologic
TB	Tuberculosis	Respiratory
URI	Upper respiratory infection	Respiratory
UTI	Urinary tract infection	Renal
VSD	Ventricular septal defect	Cardiovascular
WPW	Wolff-Parkinson-White syndrome	Cardiovascular

COMMON ABBREVIATIONS USED ON ABSTRACTS

A

A,	ant, anterior
Abc	antibiotics
abd	abdomen, abdominal
ABG	air/bone gap
abnl, abn	abnormal
A.B.P.	atrial blood pressure
abs	absent
abx	antibiotics
a.c.	before meals
adeno CA	adenocarcinoma
ADH	antidiuretic hormone
ADL	activities of daily living
adj	adjacent
adm	admission
A.F. or A. Fib	atrial fibrillation
AFP	alpha feto protein
A. flutter	atrial flutter
Ag	antigen
AIHA	autoimmune hemolytic anemia
alcoh	alcohol
ALL	acute lymphocytic leukemia
ALS	amyotrophic lateral sclerosis
AMA	against medical advice
amb	ambulatory
AMI	acute myocardial infarction
amp	amputation
A/P	assessment and plan
appl'y	apparently
arrhy	arrhythmia
asp	aspiration
aut	autopsy

B

B	bilateral
BAD	Bipolar Affective Disorder
Ba	barium
B9	benign
BE	barium enema
BID	2 x's a day
bil	bilateral
BKA	below knee amputation
BM	bone marrow, bowel movement
BP	blood pressure
BMT	bone marrow transplant
BRBPR	bright red blood per rectum
BS	bowel sounds, breath sounds, bone scan
BSO	bilateral salpingo-oophorectomy
BT	bladder tumor
BV	blood volume
Bx	biopsy

C

CA	cancer
CABG	coronary artery bypass graft
CAD	coronary artery disease
CBC	complete blood count
CBD	common bile duct
CC	chief complaint
c/c/e	clubbing, cyanosis or edema
C&DB	cough and deep breathe
CHD	congenital heart disease
C.I.S.	carcinoma in situ
CLL	chronic lymphocytic leukemia
CN II-XII	cranial nerves 2-12
CPAP	continuous positive airway pressure
CPM	continue present management
Cr	creatine
C-spine	cervical spine
C&S	culture & sensitivities
CT	computerized tomography
c/w	consistent with
CXR	chest x-ray
cysto	cystoscopy
cyt	cytology
CT C/A/P	CT scan of chest, abdomen & pelvis OK to list as CT CAP:

D

DC	discontinued
d/c	discharge
decr	decrease (d)
dep	depression
diff	differentiated
D.O.E.	dyspnea on exertion
DRE	digital rectal exam
D.T.s	delerium tremens
dx	diagnosis
dz	disease

E

EGD	esophagogastro-duodenoscopy
enlgd	enlarged
ER	estrogen receptor
ERCP	endoscopic retrograde
et	and
ETOH	alcohol
EUA	exam under anesthesia
exc('d)	excise(d)
exp lap	exploratory laparotomy
ext	extension, extended, extending

F

FB	foreign body
ff	force fluids
FH	family history
FNA	fine needle aspiration
FNAB	fine needle aspiration biopsy
FND	functional neck dissection

F/O	fiberoptic
FOB	fiberoptic bronchoscopy
FOE	fiberoptic exam
FOM	floor of mouth
FROM	full range of motion
FS	frozen section
FTSG	full thickness skin graft
F/U	follow up
FUO	fever of unknown origin
Fx	fracture

G

G	gravida
GB	gallbladder
GI	gastrointestinal
Gr	grade
GSW	gunshot wound
GT	granulation tissue
gtt	drop
gtts	drops
GU	genitourinary

H

HA	headache
HCG	human chorionic gonadotropin
HEENT	head, eyes, ears, nose & throat
HH	hiatal hernia
H/O	history of
HOB	head of bed
HPI	history of present illness
HS	hours of sleep (at bedtime)
HTN	hypertension
H&P	history and physical examination
hx	history
hyst	hysterectomy

I

I & D	incision & drainage
IHC	immunohistochemical
IMA	internal mammary artery
incr	increase (d)
ing	inguinal
inv	invade(s), invading, nvasion
invl	involve(s)(ment)(ing)
IV	intravenous
IVC	inferior vena cava
IVF	IV fluids
IVP	Intravenous pyelogram

L

L	left
lat	lateral
LE	lower extremity
lg	large
LIQ	lower inner quadrant
LLL	left lower lobe
LLQ	left lower quadrant
LN	lymph node
LND	lymph node dissection
LOQ	lower outer quadrant
LP	lumbar puncture
LS scan	liver/spleen scan
L-spine	lumbar spine
LS	lumbosacral
LUL	left upper lobe
LUQ	left upper quadrant

M

malig	malignant
mand	mandible, mandibular
max	maximum, maxillary
med	medial, mediastinoscopy
mets	metastasis, metastatic
MD	moderately differentiated
mod	moderate, moderately
MSB	main stem bronchus
MRI	magnetic resonance imaging
MRM	modified radical mastectomy
mult	multiple
MVA	motor vehicle accident

N

NA	not applicable
NED	no evidence of disease
neg	negative
NER	no evidence of recurrence
NKDA	no known drug allergies
nl	normal
nos	not otherwise specified
NR	not recorded
NSF	no significant findings
N&V	nausea & vomiting

O

obst	obstruct (-ing, -ion)
OMP	other medical problem
OSH	outside hospital

P

P	post posterior
PA	pulmonary artery, posterior-anterior
palp	palpated, palpable
P&A	percussion & auscultation
PE	physical examination
perc	percutaneous
PD	poorly differentiated
pos	positive
poss	possible
PMH	past medical history
PPD	packs per day
PR	progesterone receptor
prob	probable, probably
pt	patient
PTA	prior to admission

Q

QID	4 x's a day
QOD	every other day
quad	quadrant
qh	every hour

qhs	at bedtime
q	every
qd	every day

R

R	right
RCM	right costal margin
resec	resection, resected
RIA	radio immune assay
RLL	right lower lobe
RLQ	right lower quadrant
R/O	rule out
ROS	review of (outside slides)
RUL	right upper lobe
RUQ	right upper quadrant
RT	radiation therapy
RND	radical neck nessection
Rx	prescription (or treatment)

S

SB	small bowel
SBO	small bowel obstruction
SCCA	squamous cell carcinoma
SC(V)	supraclavicular
sm	small
SOB	shortness of breath
S/P	status post
spec	specimen
S&S	signs and symptoms
ST	soft tissue
STSG	split thickness skin graft
susp	suspicious, suspected
SV	seminal vesicles
SVC	superior vena cava
Sx	symptoms

T

T	thoracic
TAH	total abdominal hysterectomy
TNM	T = tumor primary site stageN = nodal stage (regional)M = distant metastasis stage
TS	tumor size
T-spine	thoracic spine
TVC	true vocal cord
Tx	treatment

U, V, W,

UE	upper extremity
UGI	upper gastrointestinal
undiff	undifferentiated
unk	unknown
vag	vaginal
WD	well differentiated
wnl	within normal limits
wt	weight
w/u	work-up

SYMBOLS

+	plus, positive
&	and
-	ess, minus, negative
<	less than
>	more or greater than
x	times
#	pounds, number
@	at
w/	with
w/o	without

PARTIAL DRUG LIST

Common Trade Name	Generic Name	Diseases
Accolate	zafirlukast	Prophylaxis, Chronic Treatment of Asthma
Accupril	quinapril	Hypertension
Actos	pioglitazone	Lower Blood Glucose
Achromycin	tetraacycline	Treatment of Infections
Adalat	nifedipine	Hypertension
Aerobid	flunisolide	Asthma
Aldactone	spironolactone	Congestive Heart Failure,
Altace	ramipril	Hypertension
Amaryl	glimepiride	Lower Blood Glucose
Amoxil	amoxicillin	Treatment of Infections
Apresoline	hydralazine	Hypertension
Atacand	candesartan	Hypertension
Avandia	rosiglitazone	Lower Blood Glucose
Avapro	irbesartan	Hypertension
Axid	nizatidine	Heartburn, Acid Indigestion, Sour Stomach
Azmacort	Triamcinolone acetonide	Asthma
AZT	azathioprine	Rejection in Renal Transplantation, Rheumatoid Arthritis
Beclovent	beclomethasone dipropionate	Asthma
Biaxin	clarithromycin	Treatment of Infections
Brethaire	terbutaline	Reversal of Bronchospasms
Bumex	bumetanide	Edema, Heart Failure, Hepatic & Renal Disease
Calan	verapamil	Angina, Arrhythmias
Capoten	captopril	Hypertension
Carafate	sucralfate	Duodenal Ulcer
Cardizem	diltiazem	Hypertension
Celexa	citulopram	Depression
Combivent	ipratropium	Chronic Obstructive Pulmonary Disease
Coreg	carvedilol	Congestive Heart Failure,
Covera	verapamil	Hypertension, Angina
Cozaar	losartan	Hypertension
Cytotec	misoprostol	Prevention of NSAID Gastric Ulcers, Concomitant Debilitating Disease
Demadix	torsemide	Edema, Congestive Heart Failure, Renal Disease, Hepatic Disease
Depakote	divalproex Sodium delayed-rel	Bipolar Disorder
Desyrel	trazadone	Depression
Diabinese	chlorpropamide	Hyperglycemia, to Lower Blood Glucose
DiBeta	glyburide	Lower Blood Glucose
Dilacor	diltiazem	Hypertension
Diltia	diltiazem	Hypertension
Diovan	valsartan	Hpertension
Diuril	chlorothiazide	Hypertension
Effexor XR	venlafaxine ext-rel	Depression
Elavil	amitriptyline	Depression
Flagyl	metronidazole	symptomatic trichomoniasis
Flovent	fluticasone propionate	Asthma
Glucophage	metformin	Lower Blood Glucose
Glucotrol	glipizide	Control Hyperglycemia
Glynase	glyburide	Lower Blood Glucose
Glyset	miglitol	Lower Blood Glucose
Inderal	propranolol	HTN, Angina Pectoris, MI., Migraine, Hypertrophic Subaortic Stenosis
Intal	cromolyn sodium	Bronchial Asthma
Isoptin	verapamil	Hypertension

Common Trade Name	Generic Name	Diseases
Isordil	isosorbide dinitrate	Angina Pectoris, Coronary Heart Disease
Lanoxin	digoxin	Heart Failure, Atrial Fibrillation
Lasix	furosemide	Edema, Hypertension
Levatol	penbutolol	Arterial Hypertension
Lithium	lithium carbonate	Bipolar Disorder, Manic (DSM-III)
Lopressor	metoprolol	Hypertension
Lotensin	benazepril	Hypertension
Maalox Plus	Magnesium hydrox, alum. hydrox.	Upset Stomach
Maxair	pirbuterol	Reversal of Bronchspasms
Metrogel	metronidazole	Symptomatic Trichomoniasis
Micronase	glyburide	Lower Blood Glucose
Monopril	fosinopril	Hypertension
Mylanta Double Strength	simethiconde antacids	Acid Indigestion, Sour Stomach
Nasalcrom	sulfasalazine	Allergic Rhinitis
Norvasc	amoldipine	Hypertension, Chronic Stable Angina, Vasospastic Angina
Orinase	tolbutamide	Lower Blood Glucose
Pepcid	famotidine	Duodenal & Benign Gastric Ulcers, Gastroesophageal Reflux
Pepto Bismol	bismuth subslicylate	Upset Stomach & Diarrhea
Prandin	repaglinide	Lower Blood Glucose
Precose	acarbose	Lower Blood Glucose
Prilosec	omeprazole	Duodenal Ulcer
Prinivil	lisinopril	Hypertension, Heart Failure, Acute Myocardial Infarction
Procardia	nifedipine	Vasospastic Angina, Chronic Stable Angina
Proventil	albuterol	Bronchospasms, Airway Disease,
Prozac	fluoxetine	Depression
Pulmicort Turbuhaler	budesonide	Asthma
Rezulin	troglitazone	Lower Blood Glucose
Serevent	salmeterol	Asthma
Slo-Bid Gyrocaps	theophylline	Asthma, Reversible Bronchospasms, Chronic Bronchitis, Emphysema
Sorbitrate		Angina, Coronary Artery Disease
Sular	nisoldipine	Hypertension
Tagament	cimetidine	Short Term Duodenal Ulcer
Theo-Dur	theophylline	Chronic Asthma, Chronic Lung Disease, Emphysema
Tiamate	diltiazem	Hypertension
Tiazac	diltiazem	Hypertension
Tilade	nedocromil sodium	Bronchial Asthma
Tolinase	tolazamide	Lower Blood Glucose
Tornalate	bitolterol mesylate	Bronchial Asthma, Reversible Bronchospasms
Unidur	theophylline	Chronic Asthma, Chronic Lung Disease, Emphysema
Vanceril	beclomethasone dipropionate	Bronchial Asthma
Vasotec	enalapril	Hypertension
Ventolin	albuterol	Bronchospasms, Airway Disease,
Verelan	verapamil	Hypertension
Wellbutrin	bupropion	Depression
Zantac	ranitidine	Short Term Duodenal Ulcer, Maintenance of Ulcer, Gastric Ulcer
Zebeta	bisoprolol	Hypertension
Zestril	lisinopril	Hypertension, Heart Failure, Acute Myocardial Infarction
Zoloft	sertraline	Depression
Zyflo	zileuton	Prophylaxis, Chronic Treatment of Asthma

Generic Name	Common Trade Name	Diseases
acarbose	Precose	Lower Blood Glucose
albuterol	Proventil	Bronchospasms, Airway Disease,
albuterol	Ventolin	Bronchospasms, Airway Disease,
amitriptyline	Elavil	Depression
amoldipine	Norvasc	Hypertension, Chronic Stable Angina, Vasospastic Angina
amoxicillin	Amoxil	Treatment of Infections
azathioprine	AZT	Rejection in Renal Transplantation, Rheumatoid Arthritis
beclomethasone dipropionate	Beclovent	Asthma
beclomethasone dipropionate	Vanceril	Bronchial Asthma
benazepril	Lotensin	Hypertension
bismuth subslicylate	Pepto Bismol	Upset Stomach & Diarrhea
bisoprolol	Zebeta	Hypertension
bitolterol mesylate	Tornalate	Bronchial Asthma, Reversible Bronchospasms
budesonide	Pulmicort Turbuhaler	Asthma
bumetanide	Bumex	Edema, Heart Failure, Hepatic & Renal Disease
bupropion	Wellbutrin	Depression
candesartan	Atacand	Hypertension
captopril	Capoten	Hypertension
carvedilol	Coreg	Congestive Heart Failure,
chlorothiazide	Diuril	Hypertension
chlorpropamide	Diabinese	Hyperglycemia, to Lower Blood Glucose
cimetidine	Tagament	Short Term Duodenal Ulcer
citulopram	Celexa	Depression
clarithromycin	Biaxin	Treatment of Infections
cromolyn sodium	Intal	Bronchial Asthma
digoxin	Lanoxin	Heart Failure, Atrial Fibrillation
diltiazem	Cardizem	Hypertension
diltiazem	Dilacor	Hypertension
diltiazem	Diltia	Hypertension
diltiazem	Tiamate	Hypertension
diltiazem	Tiazac	Hypertension
divalproex Sodium delayed-rel	Depakote	Bipolar Disorder
enalapril	Vasotec	Hypertension
famotidine	Pepcid	Duodenal & Benign Gastric Ulcers, Gastroesophageal Reflux
flunisolide	Aerobid	Asthma
fluoxetine	Prozac	Depression
fluticasone propionate	Flovent	Asthma
fosinopril	Monopril	Hypertension
furosemide	Lasix	Edema, Hypertension
glimepiride	Amaryl	Lower Blood Glucose
glipizide	Glucotrol	Control Hyperglycemia
glyburide	DiBeta	Lower Blood Glucose
glyburide	Glynase	Lower Blood Glucose
glyburide	Micronase	Lower Blood Glucose
hydralazine	Apresoline	Hypertension
ipratropium	Combivent	Chronic Obstructive Pulmonary Disease
irbesartan	Avapro	Hypertension
isosorbide dinitrate	Isordil	Angina Pectoris, Coronary Heart Disease
lisinopril	Prinivil	Hypertension, Heart Failure, Acute Myocardial Infarction
lisinopril	Zestril	Hypertension, Heart Failure, Acute Myocardial Infarction
lithium carbonate	Lithium	Bipolar Disorder, Manic (DSM-III)
losartan	Cozaar	Hypertension
Magnesium hydrox, alum. hydrox.	Maalox Plus	Upset Stomach

Generic Name	Common Trade Name	Diseases
metformin	Glucophage	Lower Blood Glucose
metoprolol	Lopressor	Hypertension
metronidazole	Flagyl	symptomatic trichomoniasis
miglitol	Glyset	Lower Blood Glucose
misoprostol	Cytotec	Prevention of NSAID Gastric Ulcers, Concomitant Debilitating Disease
nedocromil sodium	Tilade	Bronchial Asthma
nifedipine	Adalat	Hypertension
nifedipine	Procardia	Vasospastic Angina, Chronic Stable Angina
nisoldipine	Sular	Hypertension
nizatidine	Axid	Heartburn, Acid Indigestion, Sour Stomach
nortiptyline	pamalor	Endogenous Depression
omeprazole	Prilosec	Duodenal Ulcer
penbutolol	Levatol	Arterial Hypertension
pioglitazone	Actos	Lower Blood Glucose
pirbuterol	Maxair	Reversal of Bronchospasms
propranolol	Inderal	HTN, Angina Pectoris, MI., Migraine, Hypertrophic Subaortic Stenosis
quinapril	Accupril	Hypertension
ramipril	Altace	Hypertenion
ranitidine	Zantac	Short Term Duodenal Ulcer, Maintenance of Ulcer, Gastric Ulcer
repaglinide	Prandin	Lower Blood Glucose
rosiglitazone	Avandia	Lower Blood Glucose
salmeterol	Serevent	Asthma
sertraline	Zoloft	Depression
simethiconde antacids	Mylanta Double Strength	Acid Indigestion, Sour Stomach
spironolactone	Aldactone	Congestive Heart Failure,
sucralfate	Carafate	Duodenal Ulcer
sulfasalazine	Nasalcrom	Allergic Rhinitis
terbutaline	Brethaire	Reversal of Bronchospasms
tetraacycline	Achromycin	Treatment of Infections
theophylline	Slo-Bid Gyrocaps	Asthma, Reversible Bronchospasms, Chronic Bronchitis, Emphysema
theophylline	Theo-Dur	Chronic Asthma, Chronic Lung Disease, Emphysema
theophylline	Unidur	Chronic Asthma, Chronic Lung Disease, Emphysema
tolazamide	Tolinase	Lower Blood Glucose
tolbutamide	Orinase	Lower Blood Glucose
torsemide	Demadix	Edema, Congestive Heart Failure, Renal Disease, Hepatic Disease
trazadone	Desyrel	Depression
Triamcinolone acetonide	Azmacort	Asthma
troglitazone	Rezulin	Lower Blood Glucose
valsartan	Diovan	Hypertension
venlafaxine ext-rel	Effexor XR	Depression
verapamil	Calan	Angina, Arrhythmias
verapamil	Covera	Hypertension, Angina
verapamil	Isoptin	Hypertension
verapamil	Verelan	Hypertension
zafirlukast	Accolate	Prophylaxis, Chronic Treatment of Asthma
zileuton	Zyflo	Prophylaxis, Chronic Treatment of Asthma
	Sorbitrate	Angina, Coronary Artery Disease
